# Accuracy of Static, Dynamic, and Robotic Guided Surgery in Immediate Implant Placement: A Systematic Review and Network Meta‐Analysis

**DOI:** 10.1111/clr.70100

**Published:** 2026-02-19

**Authors:** Paolo Nava, Hamoun Sabri, Parham Hazrati, Carlo Nava, Muhammad H. A. Saleh, Hom‐Lay Wang

**Affiliations:** ^1^ Department of Periodontics and Oral Medicine University of Michigan School of Dentistry Ann Arbor Michigan USA; ^2^ Periodontics and Oral Implantology Advanced Computing and Artificial Intelligence (PICO.AI) Ann Arbor Michigan USA; ^3^ Department of Biomedical, Dental, and Morphological and Functional Imaging Sciences University of Messina Messina Italy

**Keywords:** computer‐assisted surgery, dental implants, dynamic navigation, immediate implantation, meta‐analysis, robotic surgical procedures, surgical templates, systematic review, tooth extraction, treatment outcome

## Abstract

**Objectives:**

To evaluate the accuracy of static, dynamic, and robotic computer‐assisted protocols in immediate implant placement (IIP), compared to freehand (FH) techniques.

**Materials and Methods:**

A systematic search was conducted for studies published through March 2025. Clinical studies on Computer‐Aided Implant Surgery (CAIS), reporting angular and/or linear deviations between the planned and actual implant position after IIP were included. Eligible designs comprised randomized controlled trials (RCTs), non‐randomized controlled trials (nRCTs), prospective/retrospective cohort studies (Retro), and prospective/retrospective case series (PCS/RCS). A network meta‐analysis was performed to assess deviations in implant angulation, platform, and apex positions among FH, half‐guided static (HG‐sCAIS), fully‐guided static (FG‐sCAIS), dynamic (dCAIS), and robotic (rCAIS) protocols. Covariates such as jaw, site position, study design, and risk of bias were included. Two post hoc sensitivity analyses were also conducted.

**Results:**

Eighteen studies encompassing 780 immediately placed implants were analyzed (6 RCTs; 2 PCS; 7 RCS; 3 Retro). Compared with FH, every guided protocol significantly enhanced accuracy. Mean angular deviation decreased by 3.36° with rCAIS, 2.66° with dCAIS, 1.73° with HG‐sCAIS, and 1.85° with FG‐sCAIS (*p < 0.05*). Corresponding reductions in platform deviation were 0.68, 0.71, 0.27, and 0.54 mm, while apex deviation decreased by 1.43, 1.32, 1.23, and 0.81 mm, respectively (*p < 0.05*). rCAIS and dCAIS ranked highest across all metrics.

**Conclusion:**

Within the limitations of this study, guided surgery significantly improves the accuracy of immediate implant placement. rCAIS and dCAIS demonstrated the most favorable accuracy profiles, although sensitivity analyses restricted to higher‐quality evidence suggest that differences among guided protocols may be less pronounced.

## 1 Introduction

To meet the growing demand for optimized and time‐efficient treatment protocols, immediate implant placement (IIP) has increasingly emerged as a viable alternative to delayed and early implant placement (Ickroth et al. 2024; Sabri et al. 2024). IIP offers significant advantages, including reduced overall treatment duration, fewer surgical interventions, and high patient satisfaction (Chen and Buser 2014; Tonetti et al. 2017). However, achieving optimal results with IIP requires strict adherence to biologically driven principles, as the healing dynamics of post‐extraction sites present unique challenges compared to healed ridges (Sabri et al. 2023). While expedited treatment is appealing, clinical success is ultimately determined by long‐term functional and aesthetic outcomes rather than the speed of therapy (Donos et al. 2021; Natale et al. 2024).

A key determinant of long‐term success in implant therapy, particularly in IIP, is the accuracy of implant positioning. Precise three‐dimensional (3D) placement is crucial for maintaining peri‐implant tissue stability, optimizing load distribution, and ensuring a favorable prosthetic outcome (Tahmaseb et al. 2014; Van Assche et al. 2012). Improper positioning can compromise esthetics, functional integration, and long‐term implant survival, particularly in the anterior maxilla where soft tissue recession and buccal bone loss can lead to unfavorable outcomes (Cooper 2015; Sabri et al. 2023; Wu et al. 2023). Therefore, meticulous planning and guided surgical approaches have become essential to improve predictability and minimize deviations from the planned implant position.

Guided surgery has emerged as the most advanced and reliable approach to achieve precise implant placement. By incorporating preoperative digital planning with intraoperative guidance, guided implant surgery allows for a prosthetically driven approach, minimizing human error and improving surgical accuracy (Pedrinaci et al. 2024). Compared to freehand implant placement (FH), guided techniques have been shown to reduce deviations in implant angulation, depth, and position, which are critical factors for achieving optimal functional and esthetic outcomes (Pozzi et al. 2016).

Several types of guided implant surgery techniques have been introduced, each with their own advantages and limitations. Static computer‐assisted implant surgery (sCAIS) uses prefabricated surgical templates to guide implant positioning but lacks intraoperative flexibility (Gargallo‐Albiol et al. 2019). Dynamic computer‐assisted implant surgery (dCAIS) allows real‐time tracking and intraoperative adjustments, improving surgical adaptability but requiring a steep learning curve (Jorba‐García et al. 2019; Pozzi et al. 2024). More recently, robotic computer‐assisted implant surgery (rCAIS) has been developed, integrating real‐time feedback with haptic guidance to enhance precision and minimize operator‐dependent variability (Bolding and Reebye 2022; Yang, Chen, et al. 2024). While several of these methods have been extensively studied for delayed implant placement, their accuracy in the context of IIP remains underexplored.

Currently, available systematic reviews and meta‐analyses focus on the accuracy of guided surgery in healed ridges, where anatomical stability facilitates predictable outcomes (Gargallo‐Albiol et al. 2019; Pellegrino et al. 2021). However, IIP presents unique challenges, mainly related to post‐extraction socket anatomy and more complex planning protocols (Parra‐Tresserra et al. 2021; Wu et al. 2020). To address this gap in the literature, this systematic review aimed to assess the accuracy of static, dynamic, and robotic‐assisted guided surgery techniques for IIP and to compare it with the one achieved through free‐hand placement.

## 2 Methods

### 2.1 Protocol and Search Strategy

In accordance with PRISMA for network meta‐analysis (NMA) guidelines (Hutton et al. 2015), this study was conceptualized as a systematic review to assess the accuracy and precision of freehand, static, dynamic, and robotic computer‐aided implant surgery in the context of IIP and compare these approaches with freehand placement. For this purpose, IIP was defined as the insertion of a dental implant directly into a fresh extraction socket at the time of tooth removal. The protocol of this study was registered in the PROSPERO database (Registration ID: CRD42024585878) prior to study initiation.

Details of the search query are presented in Table 1. Briefly, Web of Science, MEDLINE (via PubMed), Scopus, and Embase databases were searched. In addition, a manual search was conducted in the implant‐dentistry related journals listed in Table 1.

**TABLE 1:** Search strategy and selection criteria.

Focused question	In adult patients undergoing IIP, how does the accuracy of static, dynamic, or robotic computer‐assisted implant surgery compare to that of conventional free‐hand placement?	
PICOS	Population	Adults undergoing IIP within 24 h after extraction
	Intervention	Implant bed preparation and/or insertion using static (sCAIS), dynamic (dCAIS), or robotic (rCAIS) computer‐assisted implant surgery
	Comparison	Conventional freehand implant placement
	Outcome	Accuracy measured by angular deviation and linear deviations at the implant platform and apex (difference between planned and post‐operative implant position)
	Study Design	Randomized and non‐randomized controlled clinical trials; prospective and retrospective clinical studies; case series including at least five patients

Search strategy	
Web of Science	(guid* OR nav* OR robo*) AND implant AND (fresh OR socket OR immediate) AND (accuracy OR precision OR evaluation OR deviation) NOT (regeneration OR zygom* OR review)
PubMed/MEDLINE	((Guid*[tiab] OR navi* OR robo*) AND (implant[tiab]) AND (fresh[tiab] OR socket[tiab] OR immediate[tiab]) AND (accuracy OR precision OR evaluation OR deviation) NOT regeneration[tiab] NOT zygom*[tiab]) NOT review[ti]
Embase	(guid* OR nav* OR robo*) AND implant AND (fresh OR socket OR immediate) AND (accuracy OR precision OR evaluation OR deviation) NOT (regeneration OR zygom* OR review)
Scopus	TITLE‐ABS‐KEY ((guid* OR nav* OR robo*) AND (implant) AND (fresh OR socket OR immediate) AND (accuracy OR precision OR evaluation OR deviation) AND NOT (regeneration OR zygom* OR review))
Manual search	Clinical Oral Implants Research; Clinical Implant Dentistry and Related Research; International Journal of Oral and Maxillofacial Implants; International Journal of Oral Implantology; Journal of Oral Implantology; Journal of Clinical Periodontology; Journal of Periodontology; Journal of Prosthodontic Research; Journal of Oral and Maxillofacial Surgery; International Journal of Oral and Maxillofacial Surgery; International Journal of Implant Dentistry; Journal of Periodontal and Implant Science

### 2.2 PICOS Question

P—Population: Adult patients with at least one non‐restorable or failing tooth requiring extraction and IIP within 24 h.

I—Intervention: Implant osteotomy preparation and/or insertion using static (sCAIS), dynamic (dCAIS), or robotic (rCAIS) computer‐assisted implant surgery.

C—Comparison: Conventional FH implant placement.

O—Outcome: Accuracy measured by angular deviation and linear deviations at the implant platform and apex (difference between planned and post‐operative implant position).

S—Study design: Randomized controlled trials (RCTs), non‐randomized controlled trials (nRCTs), prospective or retrospective cohort studies, and prospective or retrospective case series.

The focused research question was: “In adult patients undergoing IIP, how does the accuracy of static, dynamic, or robotic computer‐assisted implant surgery compare to that of conventional free‐hand placement?”.

### 2.3 Eligibility Criteria

The following criteria were considered for eligibility:

- Studies reporting angular and/or linear deviations between the planned and actual position of immediately placed implants.

Conversely, the studies were excluded if they met the following criteria:

- Cadaver, model, or animal studies.
- Surgeries performed with 2D radiographic stents or analog guides.
- No actual insertion of the implants.
- Unclear description on accuracy measurements.
- Insufficient information on timing of implant placement after tooth extraction.
- Multiple publications on the same patient population.
- Group size not reported.

### 2.4 Study Selection

Two reviewers (P.N. and C.N.) independently screened all titles and abstracts. Additionally, the reference lists of the selected articles and the bibliographies of relevant systematic reviews were manually searched to identify further eligible studies. Any discrepancies between the reviewers were resolved through discussion or, if necessary, by consultation with a third reviewer (H.L.W.). Inter‐reviewer agreement was assessed using Cohen's kappa coefficient, yielding a value of *κ* = 0.87.

Full‐text evaluation of the articles was then carried out according to predefined inclusion and exclusion criteria.

### 2.5 Data Extraction

Studies that met the inclusion criteria underwent data extraction by two independent reviewers (P.N. and C.N.) using a standardized extraction form. The following information was collected:

1. Complete bibliographic information as well as study setting.
2. Study design (randomized controlled trials (RCTs), non‐randomized controlled trials (nRCTs), prospective and retrospective studies, case series).
3. Subject population characteristics (age, gender, number of participants, and number of implants).
4. Site characteristics (e.g., maxilla/mandible, anterior/posterior, partial or full edentulism, open‐ended or tooth‐bounded).
5. Type of guidance (sCAIS, dCAIS, rCAIS, FH). Static guided protocols were further stratified into two categories: Fully Guided static (FG‐sCAIS) and Half Guided static (HG‐sCAIS). FG‐sCAIS included cases in which both site preparation and implant insertion were performed using the surgical guide. HG‐sCAIS procedures included cases in which either the entire site preparation was guided but the implant insertion was performed freehand, or only the initial steps of osteotomy preparation were performed using the surgical guide.
6. Evaluation methods (e.g., cone beam computed tomography (CBCT) and/or superimposition of intraoral digital scans).
7. Angular and linear deviations between the planned and post‐operative implant positions.

### 2.6 Risk of Bias Assessment

The quality of the included studies was assessed independently by two authors (H.S. and P.H.) using the Cochrane Risk of Bias tool version 2 (RoB 2) for RCTs (Sterne et al. 2019), the Joanna Briggs Institute (JBI) Critical Appraisal tool for case series (Munn et al. 2023), and the Newcastle‐Ottawa Scale (NOS) for retrospective cohort studies (Wells et al. 2000). Risk of bias plots were generated using the *robvis* tool (McGuinness and Higgins 2021). Based on the assessments, studies were categorized as having low, moderate, or high risk of bias. Inter‐reviewer agreement was calculated using Cohen's Kappa. For overall risk‐of‐bias classification, we applied predefined criteria for JBI and NOS:

- JBI (Case Series): studies scoring 8–10/10 were classified as low risk, 5–7/10 as moderate risk, and ≤ 4/10 as high risk.
- NOS (Retrospective Cohorts): studies scoring 7–9/9 were classified as low risk, 5–6/9 as moderate risk, and ≤ 4/9 as high risk.

### 2.7 Data Analysis and Statistical Methodology

A network meta‐analysis was conducted to compare implant placement accuracy outcomes across five placement protocols: FH, HG‐sCAIS, FG‐sCAIS, dCAIS, rCAIS. Prior to modeling, transitivity was evaluated by ensuring that the included studies were sufficiently comparable in terms of clinical and methodological characteristics, including patient population, implant site, and follow‐up protocols. Each study arm was weighted using the inverse of the standard error (SE) derived from the reported standard deviations (SDs) and sample sizes for the outcomes. Studies lacking SDs or yielding non‐finite weights were excluded from the model. A frequentist weighted linear mixed‐effects network meta‐analysis was fitted using Restricted Maximum Likelihood (REML), incorporating a random intercept for study IDs to account for clustering within studies. The following study‐level covariates were included as fixed effects to control for potential confounding: jaw location (maxilla percentage), site location (anterior percentage), study design (RCT, case series, retrospective), and risk of bias, categorized as low, moderate, or high.

League tables were generated to facilitate pairwise comparisons across all groups. Model fit was assessed using the Akaike Information Criterion (AIC). Results are reported as mean differences (° for angular; mm for linear deviations) along with 95% Wald confidence intervals, degrees of freedom, and *p*‐values. To address multiple testing, False Discovery Rate (FDR) correction was applied to *p*‐values for all pairwise comparisons. For ranking purposes, group‐specific marginal means were extracted from the fitted models using estimated marginal means (EMMs), which reflect adjusted average deviation values accounting for all model covariates. Standard errors and 95% confidence intervals for these modeled means were also reported. These modeled means were then used to assign ranks from 1 (most accurate) to 5 (least accurate) across each of the three outcomes: angular, platform, and apex deviation. Heterogeneity was assessed based on variance components derived from the mixed‐effects models. Specifically, we reported the between‐study standard deviation (*τ*), residual (within‐study) standard deviation (*σ*), and calculated the *I* ^2^ statistic to estimate the proportion of total variability attributable to between‐study heterogeneity. To supplement the model‐based estimates, we also calculated conventional inverse‐variance weighted pooled means and 95% confidence intervals for each group and outcome.

In addition, two post‐hoc sensitivity analyses were performed using a traditional contrast‐based frequentist NMA framework (netmeta package): (1) an RCT‐only NMA, and (2) an NMA restricted to RCTs plus low‐ and moderate‐risk non‐randomized studies. For these models, pairwise comparisons, network geometry, league tables, and treatment rankings (*p*‐scores) were generated.

Transitivity across treatment nodes was further evaluated by comparing distributions of key clinical effect modifiers across groups using Kruskal–Wallis tests for continuous variables and contingency tables for categorical variables.

Consistency was assessed using design‐by‐treatment interaction models and node‐splitting (SIDE) procedures. In the RCT‐only analyses, statistical evidence of inconsistency was detected for angular deviation, whereas the apex deviation network was loop‐free and did not allow meaningful loop‐based inconsistency assessment. In the mixed‐design networks, expected design‐related differences were observed; however, the direction of treatment effects was consistent across analytical approaches.

Statistical analyses were performed by one author with experience in biostatistics (H.S.) using RStudio and the following packages: lme4 and lmerTest for mixed‐effects modeling (Bates et al. 2015; Kuznetsova et al. 2017), emmeans for contrast estimation and igraph, dplyr, and ggplot2 for network visualization and data handling.

### 2.8 Confidence in Network Meta‐Analysis (CINeMA) Assessment

The confidence in the results of the network meta‐analysis was evaluated using the CINeMA (Confidence in Network Meta‐Analysis) framework (Nikolakopoulou et al. 2020), which follows the GRADE principles tailored to NMA. CINeMA assesses the confidence in each treatment comparison across six domains: within‐study bias, reporting bias, indirectness, imprecision, heterogeneity, and incoherence. The analysis was based on the network geometry, effect estimates, and their corresponding standard errors, as well as study‐level characteristics including risk of bias and study design.

Study‐level risk of bias assessments were imported into the CINeMA web application, and a summary table of confidence ratings for each pairwise comparison was generated. Confidence ratings were classified as high, moderate, low, or very low, based on the extent of concern across the six domains. This structured approach allowed for a transparent appraisal of the robustness of findings across all pairwise comparisons in the network.

## 3 Results

### 3.1 Study Selection

**FIGURE 1:**
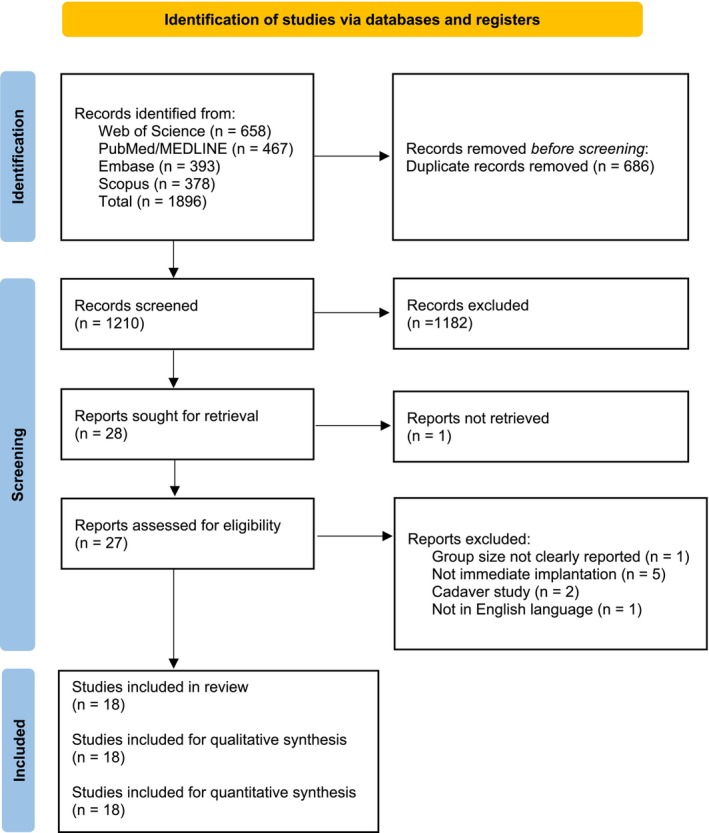
PRISMA flowchart.

The initial search across four electronic databases yielded a total of 1896 records. After removing duplicate entries, 1210 unique articles remained for title and abstract screening. Of these, 27 were selected for full‐text evaluation. Following the application of the predefined inclusion and exclusion criteria, nine studies were excluded, with the specific reasons detailed in Table S1. Consequently, 18 studies met the eligibility criteria and were included in the final analysis (Figure 1). Among these, six were RCTs (Chandran et al. 2023; Feng et al. 2022; Han et al. 2021; Kraft et al. 2020; Wei et al. 2022; Yang and Geng 2024), nine were case series (two with a prospective design (Geng et al. 2023; Mittal et al. 2024) and seven with a retrospective design (Albiero et al. 2019; Alzoubi et al. 2016; Battista et al. 2022; Li, Dai, et al. 2024; Liu et al. 2025; Yang, Xu, et al. 2024; Zhao et al. 2024)), and three were retrospective cohort studies (Chen et al. 2020; Geng et al. 2024; Li, Zhao, et al. 2024) (Table 2).

**TABLE 2:** Included studies.

Study	Design	Protocol	Patients	Implants	Maxillary implants	Mandibular implants	Anterior implants	Posterior implants	Full arch	Open‐ended sites	Guide support	Angular deviation (°)	Platform deviation (mm)	Apex deviation (mm)
Albiero et al. 2019	RCS	FG‐sCAIS	10	60	42	18	—	—	Y	Y	Mucosa pins	3.42 ± 1.52	1.28 ± 0.59	1.65 ± 0.71
Alzoubi et al. 2016	RCS	FG‐sCAIS	20	25	—	—	8	17	N	N	Teeth	3.49 ± 2.46	0.85 ± 0.65	1.05 ± 0.65
Battista et al. 2022	RCS	dCAIS	12	22	22	0	22	0	N	—	—	2.50 ± 0.41	0.77 ± 0.25	1.2 ± 0.61
Chandran et al. 2023	RCT	FG‐sCAIS	29	40	19	21	—	—	N	—	Teeth	0.83 ± 0.53	—	—
		FH	32	40	18	22	—	—	N	—	—	6.09 ± 3.23	—	—
Chen et al. 2020	Retro	FG‐sCAIS	13	17	14	3	17	0	N	N	Teeth	1.69 ± 0.94	0.66 ± 0.26	0.96 ± 0.41
		HG‐sCAIS	19	23	20	3	23	0	N	N	Teeth	2.57 ± 1.57	1.1 ± 0.76	1.43 ± 0.70
Feng et al. 2022	RCT	FG‐sCAIS	20	20	20	0	20	0	N	N	Teeth pins	3.07 ± 2.18	0.99 ± 0.63	1.5 ± 0.75
		dCAIS	20	20	20	0	20	0	N	N	Teeth pins	3.23 ± 1.67	1.06 ± 0.55	1.18 ± 0.53
Geng et al. 2024	Retro	dCAIS	28	40	0	40	0	40	N	Y	Teeth	0.88 ± 0.45	0.55 ± 0.08	0.52 ± 0.13
		FG‐sCAIS	26	44	0	44	0	44	N	Y	Teeth	1.77 ± 0.3	0.73 ± 0.10	1.33 ± 0.42
		FH	30	46	0	46	0	46	N	Y	Teeth	3.52 ± 1.03	1.28 ± 0.12	2.22 ± 0.3
Geng et al. 2023	PCS	dCAIS	8	11	11	0	11	0	N	N	—	1.48 ± 0.91	0.76 ± 0.08	1.11 ± 0.18
Han et al. 2021, ^a^	RCT	HG‐sCAIS	30	50	50	0	50	0	N	—	Teeth	2.17 ± 0.92	0.74 ± 0.21	0.81 ± 0.16
Kraft et al. 2020	RCT	HG‐sCAIS	12	12	12	0	12	0	N	N	Teeth	3.6 ± 2.84	1.34 ± 0.99	1.97 ± 1.04
		FG‐sCAIS	12	12	12	0	12	0	N	N	Teeth	5.36 ± 4.53	1.26 ± 0.57	2.5 ± 1.67
Li, Dai, et al. 2024	Retro	FH	30	40	33	7	33	0	N	N	—	6.46 ± 2.21	1.29 ± 0.52	1.78 ± 0.59
		HG‐sCAIS	18	33	22	11	22	0	N	N	Teeth	2.94 ± 1.71	1.01 ± 0.41	1.24 ± 0.52
		rCAIS	21	33	22	11	22	0	N	N	—	1.46 ± 0.57	0.62 ± 0.28	0.65 ± 0.27
Li, Zhao, et al. 2024 (2)	RCS	rCAIS	20	20	16	4	20	0	N	N	—	1.27 ± 0.47	0.71 ± 0.27	0.69 ± 0.26
Liu et al. 2025	RCS	rCAIS	8	8	—	—	—	—	N	Y	—	1.98 ± 2.7	0.83 ± 0.52	0.97 ± 0.64
Mittal et al. 2024	PCS	HG‐sCAIS	12	12	4	8	0	12	N	N	Teeth	1.03 ± 0.7	0.26 ± 0.3	0.23 ± 0.24
Wei et al. 2022	RCT	FH	12	12	12	0	12	0	N	N	—	5.97 ± 5.37	1.51 ± 0.67	1.94 ± 0.86
		dCAIS	12	12	12	0	12	0	N	N	—	2.51 ± 1.5	1.01 ± 0.41	0.88 ± 0.43
Yang, Chen, et al. 2024	RCT	FH	32	50	50	0	0	50	N	Y	—	3.35 ± 1.12	1.26 ± 0.13	1.33 ± 0.42
		dCAIS	28	46	46	0	0	46	N	Y	—	1.03 ± 0.55	0.56 ± 0.07	0.49 ± 0.26
Yang, Xu, et al. 2024 (2)	RCS	rCAIS	12	12	2	10	0	12	N	Y	—	1.05 ± 0.55	0.46 ± 0.15	0.46 ± 0.14
Zhao et al. 2024	RCS	rCAIS	15	20	20	0	20	0	N	N	—	1.17 ± 0.73	0.75 ± 0.20	0.7 ± 0.27

Abbreviations: PCS, prospective case series; RCS, retrospective case series; RCT, randomized controlled trial; Retro, retrospective cohort.

^a^ The study also included a second arm that was excluded due to the use of an analog surgical guide.

### 3.2 Studies Characteristics, Interventions and Comparisons

The selected studies collectively reported on 780 immediately placed implants across a total of 541 patients included in the analysis. The distribution of implants and patients for the five surgical techniques examined was as follows: a total of 108 patients received 151 implants using dCAIS, while the rCAIS approach was used in 76 patients for the placement of 93 implants. The FG‐sCAIS was the most widely used protocol, adopted in 130 patients with a total of 218 implants placed. The HG‐sCAIS protocol was applied in 91 patients for 130 implants, whereas the FH protocol, representing the control group in several studies, involved 136 patients and accounted for 188 implants.

Only one study involved IIP in arches that became fully edentulous following extractions (Albiero et al. 2019). Additionally, five studies included implants placed in open‐ended sites, although these represented only a portion of the implant cases. In all studies involving sCAIS, the surgical guides were exclusively tooth‐supported, with two exceptions: one study utilized a tooth‐supported guide with fixation pins (Feng et al. 2022), while another employed a mucosa‐supported guide with fixation pins (Albiero et al. 2019).

Various digital planning software and implant systems were employed across the included studies, depending on the type of guided or free‐hand surgical protocol applied. In the dCAIS group, the following software platforms were used: DTX Studio/X Guide, Digital‐care, Iris‐100, and Nobel Clinician. The implant systems used in this group included: Nobel, Straumann, Neodent, Megagen, Anthogyr, and Denton (Battista et al. 2022; Feng et al. 2022; Geng et al. 2024, 2023; Wei et al. 2022; Yang and Geng 2024). In the FG‐sCAIS static surgical group, the software platforms employed were: Simplant, Anatomage Invivo 5, R2 Gate, Nobel Clinician, and 3Shape. The implant brands used included: Ankylos, Nobel, Megagen, Straumann, and Neodent (Albiero et al. 2019; Alzoubi et al. 2016; Chandran et al. 2023; Chen et al. 2020; Feng et al. 2022; Geng et al. 2024; Kraft et al. 2020).

Implants used in the free‐hand group included: Megagen, Nobel, and Straumann (Chandran et al. 2023; Geng et al. 2024; Li, Zhao, et al. 2024; Wei et al. 2022; Yang and Geng 2024).

In the HG‐sCAIS group, the software platforms utilized were: Simplant, CoDiagnostiX, and 3Shape. The implant systems used included: Nobel, Neodent, Osstem and DIO (Chen et al. 2020; Han et al. 2021; Kraft et al. 2020; Li, Zhao, et al. 2024; Mittal et al. 2024).

Finally, in the rCAIS group, the surgical guidance system used was Remebot. The implant brands adopted in this group were: Straumann, Astra, Nobel, and Bicon (Li, Zhao, et al. 2024; Li, Dai, et al. 2024; Liu et al. 2025; Yang, Chen, et al. 2024; Zhao et al. 2024).

All included studies assessed implant placement accuracy through the superimposition of pre‐ and postoperative CBCT scans, with the exception of Chandran et al. (2023), which utilized the superimposition of intraoral scans with scan bodies (Chandran et al. 2023).

**FIGURE 2:**
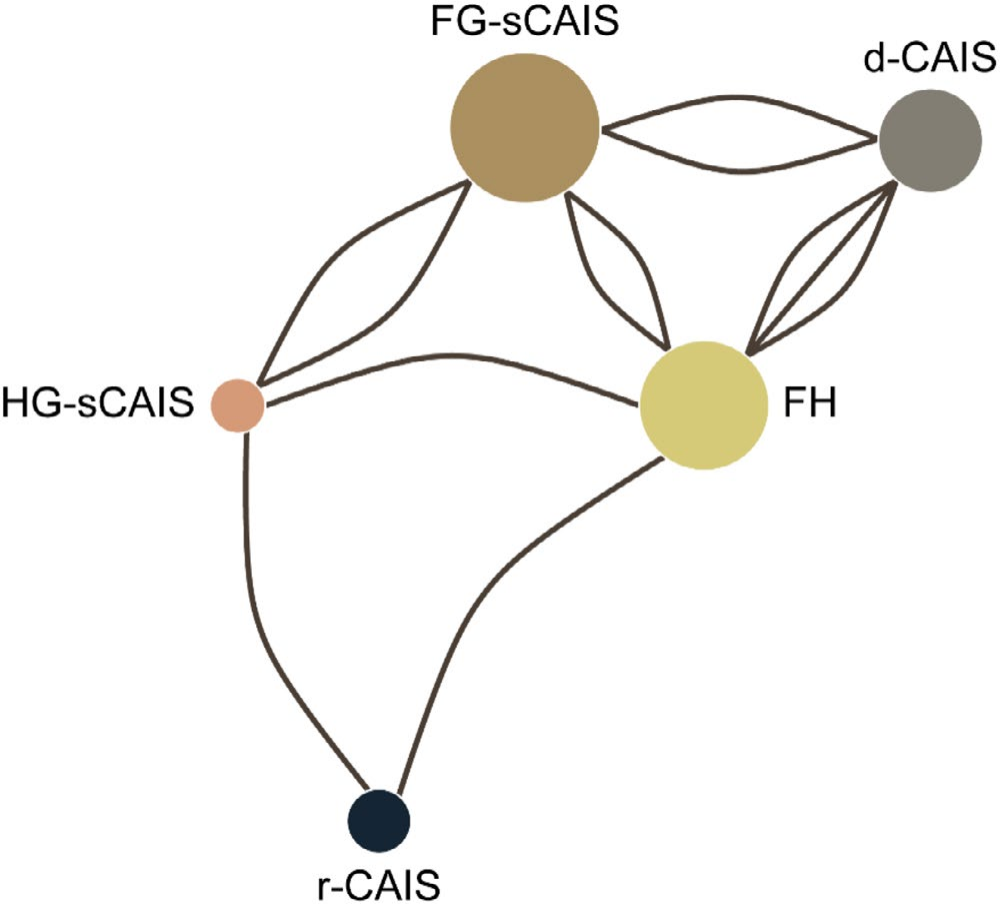
Network of direct comparisons between surgical guidance protocols. Each node represents a guidance method, with node size proportional to the total number of implants included for that method. Edges indicate the presence of at least one direct comparison between protocols: rCAIS; dCAIS; FG‐sCAIS; HG‐sCAIS; FH.

Several of the included studies were designed as comparative trials evaluating different surgical techniques within the same investigation. A comparison between FG‐sCAIS and FH was conducted (Chandran et al. 2023). FG‐sCAIS was also compared with HG‐sCAIS in two separate studies (Chen et al. 2020; Kraft et al. 2020). One study assessed the outcomes of dCAIS versus FG‐sCAIS (Feng et al. 2022), while another included a three‐arm comparison involving dCAIS, FG‐sCAIS and FH (Geng et al. 2024). Comparisons between dCAIS and FH were also explored in two separate studies (Wei et al. 2022; Yang and Geng 2024). Lastly, a three‐arm study compared FH, HG‐sCAIS, and rCAIS (Li, Zhao, et al. 2024) (Figure 2).

In contrast, several studies were case series, focusing exclusively on one technique uniformly performed across all participants. Specifically, four studies investigated rCAIS (Li, Dai, et al. 2024; Liu et al. 2025; Yang, Xu, et al. 2024; Zhao et al. 2024), two studies examined FG‐sCAIS (Albiero et al. 2019; Alzoubi et al. 2016), two were dedicated to dCAIS (Battista et al. 2022; Geng et al. 2023), and one study evaluated only HG‐sCAIS (Mittal et al. 2024) (Figure 2).

### 3.3 Risk of Bias and Quality of Included Studies

**FIGURE 3:**
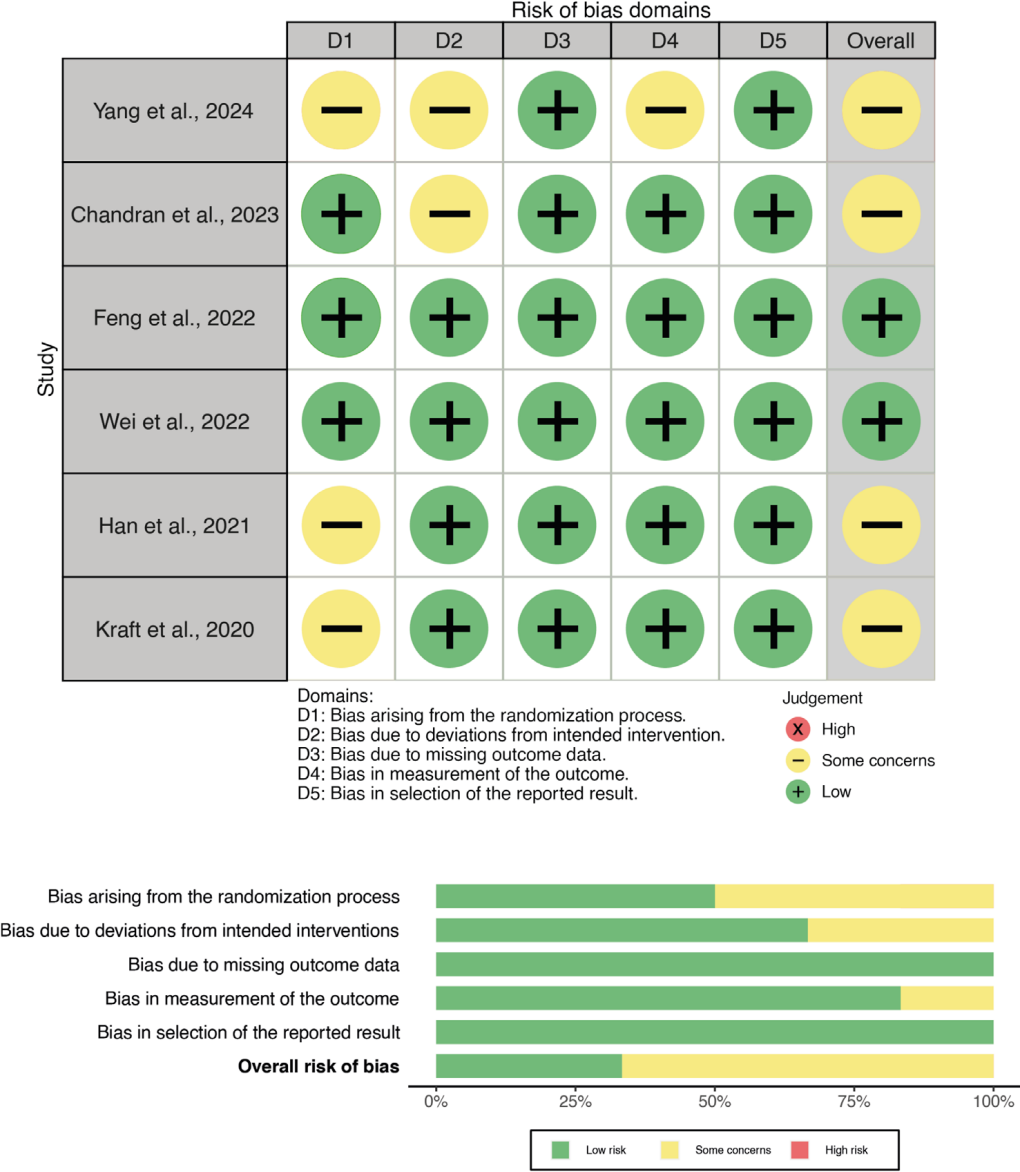
Risk of bias assessment for randomized controlled trials.

The results of the risk of bias assessment are summarized in Figure 3 for RCTs evaluated using the RoB2, and in Table 3 for case series assessed with JBI critical appraisal tool. Also, Table 4 presents the risk of bias within retrospective cohort studies, according to NOS. The inter‐examiner agreement was substantial with all tools (RoB2 = 0.85 (95% CI: 0.79–0.91); NOS = 0.81 (95% CI: 0.69–0.93); JBI = 0.82 (95% CI: 0.75–0.89)).

**TABLE 3:** The Joanna Briggs Institute (JBI) critical appraisal tool for quality assessment of case series studies.

Study	Question 1	Question 2	Question 3	Question 4	Question 5	Question 6	Question 7	Question 8	Question 9	Question 10	Design	Overall risk
Liu et al. 2025	Yes	Yes	Yes	Yes	Yes	Yes	No	Yes	Yes	Yes	Retrospective case series	Moderate
Li, Zhao, et al. 2024 (2)	Yes	Yes	Yes	Unclear	Unclear	Yes	Yes	Yes	No	Yes	Retrospective case series	Moderate
Mittal et al. 2024	Yes	Yes	Yes	Yes	No	Yes	Yes	Yes	No	Yes	Prospective case series	Moderate
Yang, Xu, et al. 2024 (2)	Yes	Yes	Yes	Yes	Unclear	Yes	Yes	Yes	Yes	Yes	Retrospective case series	Moderate
Zhao et al. 2024	Yes	Yes	Yes	Yes	Unclear	Yes	Yes	Yes	Yes	No	Retrospective case series	Moderate
Battista et al. 2022	Yes	Yes	Yes	Yes	Yes	Yes	No	Yes	Yes	No	Retrospective case series	Moderate
Albiero et al. 2019	No	Yes	Yes	Yes	Unclear	No	No	No	No	Yes	Retrospective case series	Moderate
Alzoubi et al. 2016	No	Yes	Yes	Yes	Unclear	No	No	No	No	No	Retrospective case series	Moderate
Geng et al. 2023	Yes	Yes	Yes	Unclear	Unclear	No	Yes	Yes	Yes	Yes	Prospective case series	Low

*Note:* Question 1: Were there clear criteria for inclusion in the case series? Question 2: Was the condition measured in a standard, reliable way for all participants included in the case series? Question 3: Were valid methods used for identification of the condition for all participants included in the case series? Question 4: Did the case series have consecutive inclusion of participants? Question 5: Did the case series have complete inclusion of participants? Question 6: Was there clear reporting of the demographics of the participants in the study? Question 7: Was there clear reporting of clinical information of the participants? Question 8: Were the outcomes or follow‐up results of cases clearly reported? Question 9: Was there clear reporting of the presenting sites'/clinics' demographic information? Question 10: Was statistical analysis appropriate?

**TABLE 4:** Newcastle‐Ottawa Scale (NOS) tool for quality assessment of retrospective studies.

Author, year	Domains								Total	Overall risk
	Selection				Comparability	Outcome				
	Representativeness of the exposed cohort	Selection of the non‐exposed cohort	Ascertainment of exposure	Demonstration that outcome of interest was not present at start of study	Comparability of cohorts on the basis of the design or analysis	Assessment of outcome	Was follow‐up long enough for outcomes to occur	Adequacy of follow up of cohorts		
Geng et al. 2024	*	*	*	*	**		*	*	8	Low
Li, Dai, et al. (2024)	*	*	*	*	*		*	*	7	Low
Chen et al. 2020	*	*	*	*	*		*	*	7	Low

*Note:* The asterisk denotes the Newcastle Ottawa scale system that involves the attribution of one or two stars for each domain. The total count of the stars is reported in the column total.

### 3.4 Mixed‐Effects Network Meta‐Analysis

#### 3.4.1 Angular Deviation

**FIGURE 4:**
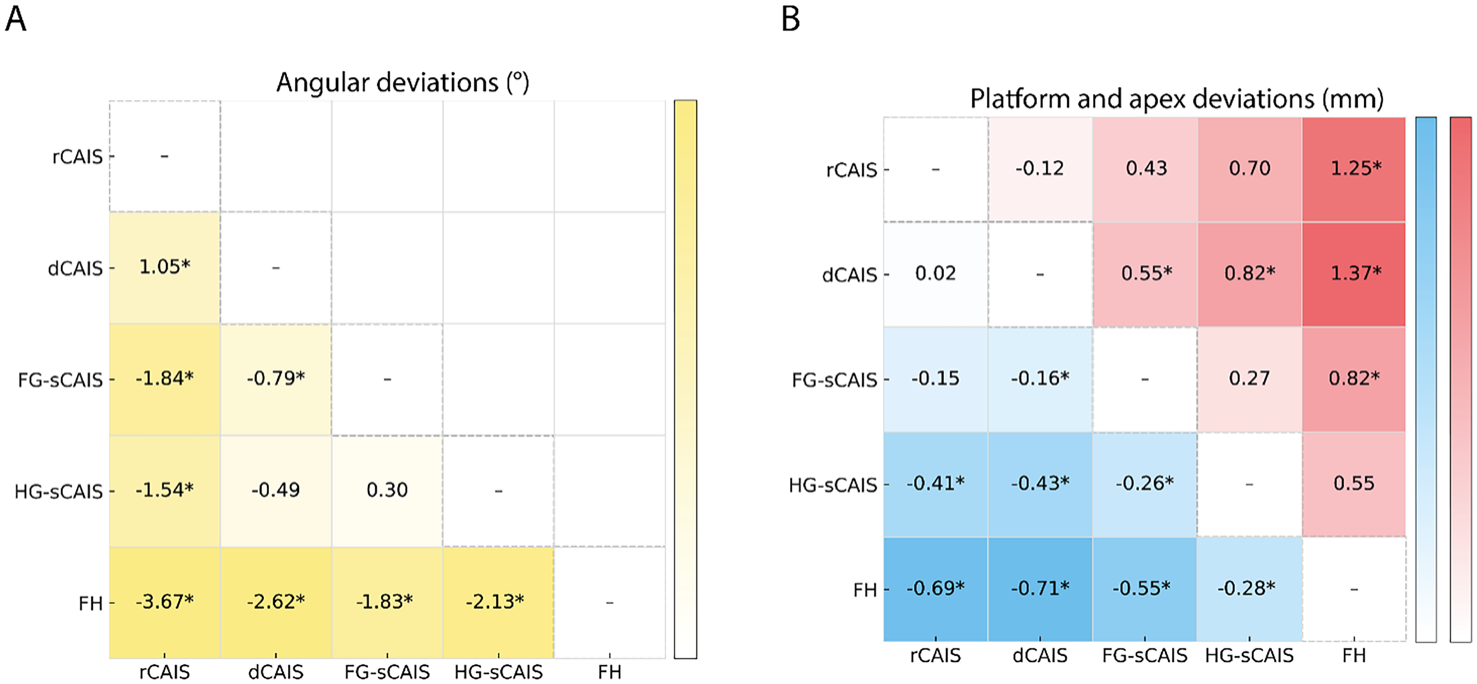
League tables comparing the accuracy of different guided implant surgery techniques with color intensity proportional to the magnitude of the deviation. Positive values favor the *y* axis; Negative values favor the *x* axis. (A) Angular deviations: The lower‐triangular matrix reports mean differences between techniques, (yellow scale). (B) Platform and apex deviations: The matrix displays pairwise differences at the implant platform (blue, lower triangle) and apex (red, upper triangle). *: Statistically significant.

According to the mixed‐effects network meta‐analysis model with FH as reference, all computer‐assisted protocols were associated with significantly lower angular deviation. Compared with FH, mean angular deviation was reduced by 3.36° with rCAIS (*p* < 0.001), 2.66° with dCAIS (*p* < 0.001), 1.85° with FG‐sCAIS (*p* = 0.001), and 1.73° with HG‐sCAIS (*p* = 0.036). Pairwise comparisons confirmed that all guided techniques produced significantly smaller angular deviations than FH. Among guided approaches, rCAIS exhibited the lowest angular deviation and was significantly more accurate than FG‐sCAIS (−1.84°; *p* < 0.001) and HG‐sCAIS (−1.54°; *p* = 0.003). dCAIS also showed significantly lower deviation than FG‐sCAIS (−0.79°; *p* < 0.001), whereas its difference from HG‐sCAIS was not statistically significant (*p* = 0.353). No significant difference was observed between FG‐sCAIS and HG‐sCAIS (0.30°; *p* = 0.5483). None of the examined study‐level covariates (risk of bias category, maxilla percentage, anterior percentage, presence of open‐ended sites, or study design) showed a statistically significant association with angular deviation (all *p* > 0.1) (Figure 4; Table 5; Table S2).

**TABLE 5:** Mixed‐effects network meta‐analysis model results for angular deviation, platform deviation and apex deviation. (Free‐hand placement was selected as the reference group).

Predictor	Estimate	SE	*p*
Angular deviation			
(Intercept)	−0.03	0.996	0.977
rCAIS	−3.357	0.55	**0.0002**
dCAIS	−2.66	0.27	**0.0004**
FG‐sCAIS	−1.854	0.28	**0.001**
HG‐sCAIS	−1.729	0.65	**0.036**
Risk of bias			
Moderate	Ref.	Ref.	Ref.
Low	−0.45	0.604	0.486
High	−1.38	0.830	0.146
Maxilla %	0.0089	0.0110	0.469
Anterior %	0.02	0.0114	0.111
Open‐ended (yes)	1.59	0.973	0.141
Study design			
RCT	Ref.	Ref.	Ref.
Case series	−1.0287	0.8541	0.268
Retrospective	−1.1707	0.7913	0.14

Platform deviation			
(Intercept)	−0.132	0.156	0.421
rCAIS	−0.684	0.07	< **0.001**
dCAIS	−0.713	0.01	< **0.001**
FG‐sCAIS	−0.543	0.01	< **0.001**
HG‐sCAIS	−0.274	0.07	**0.006**
Risk of bias			
Moderate	Ref.	Ref.	Ref.
Low	−0.076	0.088	0.411
High	−0.265	0.127	0.083
Maxilla %	−0.001	0.002	0.615
Anterior %	0.0108	0.002	**0.0008**
Open‐ended: yes	0.867	0.189	**0.0023**
Study design			
RCT	Ref.	Ref.	Ref.
Case series	−0.198	0.101	0.105
Retrospective	−0.381	0.106	0.142

Apex deviation			
(Intercept)	1.545	0.403	**0.0024**
rCAIS	−1.428	0.296	< **0.001**
dCAIS	−1.316	0.176	< **0.001**
FG‐sCAIS	−0.810	0.284	**0.014**
HG‐sCAIS	−1.229	0.328	**0.0028**
Maxilla (%)	0.007	0.006	0.299
Anterior (%)	−0.002	0.005	0.634
Open ended sites (yes)	1.414	0.566	**0.03**
Risk of bias			
Moderate	Ref.	Ref.	Ref.
Low	0.192	0.271	0.492
High	−0.812	0.321	**0.026**
Study design			
RCT	Ref.	Ref.	Ref.
Case series	−0.221	0.378	0.569
Retrospective	−0.045	0.266	0.521

*Note:* The bold values represent statistically significant *p*‐values.

#### 3.4.2 Platform Deviation

In the mixed‐effects network meta‐analysis, all computer‐assisted protocols showed significantly lower platform deviation when FH was used as reference. Relative to FH, mean platform deviation decreased by 0.71 mm with dCAIS (*p* < 0.001), 0.68 mm with rCAIS (*p* < 0.001), 0.54 mm with FG‐sCAIS (*p* < 0.001), and 0.27 mm with HG‐sCAIS (*p* = 0.006). Pairwise comparisons confirmed these findings (all *p* ≤ 0.005).

Among guided approaches, no significant difference was found between dCAIS and rCAIS (−0.017 mm; *p* = 0.853), and both yielded significantly lower platform deviation than HG‐sCAIS (−0.428 mm and −0.411 mm, respectively; *p* < 0.001). dCAIS also showed a modest but significant advantage over FG‐sCAIS (−0.165 mm; *p* < 0.001), whereas the difference between rCAIS and FG‐sCAIS was not statistically significant (*p* = 0.106). Within static protocols, FG‐sCAIS was associated with significantly lower platform deviation than HG‐sCAIS (−0.263 mm; *p* = 0.0072).

Regarding study‐level covariates, a higher proportion of anterior implants was significantly associated with increased platform deviation (0.0108 mm per 1% increase; *p* < 0.001), and the presence of open‐ended sites was likewise linked to greater deviation (+0.867 mm; *p* = 0.0023). Risk of bias category, maxillary location, and study design (RCT, case series, or retrospective) were not significantly associated with platform deviation (all *p* > 0.05) (Figure 4; Table 5; Table S2).

#### 3.4.3 Apex Deviation

In the mixed‐effects network meta‐analysis, all computer‐assisted protocols were associated with significantly lower apex deviation when FH was used as the reference. Relative to FH, apex deviation decreased by 1.43 mm with rCAIS (*p* < 0.001), 1.32 mm with dCAIS (*p* < 0.001), 0.81 mm with FG‐sCAIS (*p* = 0.014), and 1.23 mm with HG‐sCAIS (*p* = 0.003). Pairwise contrasts confirmed significantly lower apex deviation for dCAIS and rCAIS compared with FH (−1.370 and −1.250 mm, respectively; both *p* ≤ 0.0002), and FH also showed significantly higher deviation than FG‐sCAIS (0.823 mm; *p* = 0.004), whereas the difference between FH and HG‐sCAIS did not reach statistical significance (*p* = 0.175). No significant difference was observed between rCAIS and dCAIS (−0.120 mm; *p* = 0.679). dCAIS showed significantly lower apex deviation than FG‐sCAIS and HG‐sCAIS (−0.547 mm, *p* = 0.029; −0.819 mm, *p* = 0.037, respectively), while the comparisons between rCAIS and FG‐sCAIS or HG‐sCAIS were not statistically significant (*p* = 0.175 and *p* = 0.052, respectively). Apex deviation did not differ significantly between FG‐sCAIS and HG‐sCAIS (*p* = 0.482).

Regarding study‐level covariates, the presence of open‐ended sites was associated with significantly higher apex deviation (+1.414 mm; *p* = 0.03). Studies rated at high risk of bias exhibited significantly lower apex deviation than those with moderate risk (−0.812 mm; *p* = 0.026). Maxillary location, proportion of anterior implants, and study design (RCT, case series, or retrospective) were not significantly associated with apex deviation (all *p* > 0.29) (Figure 4; Table 5; Table S2).

#### 3.4.4 Treatment Rankings

The ranking of surgical protocols across all three outcomes is summarized below.

In terms of angular deviation, rCAIS achieved the highest accuracy and was ranked first (Estimate: 0.48°, SE: 0.34), followed by dCAIS (1.53°, SE: 0.22). Among static systems, HG‐sCAIS ranked third (2.02°, SE: 0.42), FG‐sCAIS fourth (2.32°, SE: 0.24), and FH was consistently the least accurate, ranking last with the highest deviation (4.15°, SE: 0.31).

For platform deviation, dCAIS again demonstrated the best performance with the lowest mean deviation (0.55 mm, SE: 0.06), followed closely by rCAIS (0.57 mm, SE: 0.09). FG‐sCAIS and HG‐sCAIS ranked third and fourth (0.72 mm and 0.95 mm, respectively), while FH once again ranked lowest with the greatest platform deviation (1.26 mm, SE: 0.06), reinforcing the superiority of guided approaches over freehand placement.

Regarding apex deviation, rCAIS was ranked first (0.45 mm, SE: 0.20), narrowly outperforming dCAIS (0.56 mm, SE: 0.13). HG‐sCAIS and FG‐sCAIS followed (0.65 mm and 1.06 mm, respectively), and FH exhibited the largest apex deviation (1.87 mm, SE: 0.19), again placing it last among all techniques evaluated.

**FIGURE 5:**
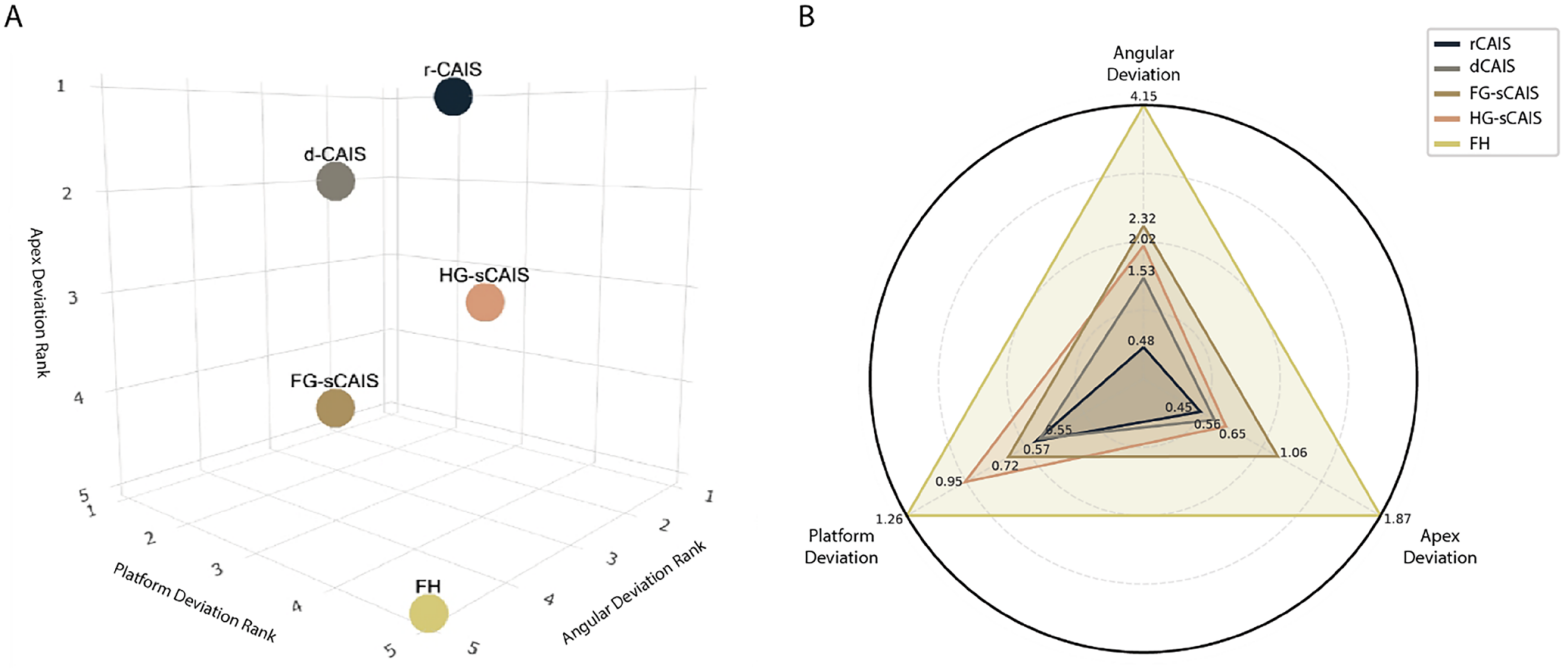
Ranking plots of implant placement protocols based on accuracy outcomes. Rankings were derived from modeled mean deviation values adjusted for confounders in a mixed‐effects network meta‐analysis. (A) 3D plot. The axes represent the relative ranking of each technique for angular deviation, platform deviation, and apex deviation (ranked from 1 = best to 5 = worst). (B) Radar plot illustrates the modeled mean deviation values (angular, platform, and apex) for each implant placement protocol. Each vertex corresponds to one of the three deviation parameters, scaled to their respective maximum values to allow visual comparability. Numerical labels indicate the means obtained from the mixed‐effects network meta‐analysis.

Across all three domains, dCAIS and rCAIS consistently ranked in the top two, while freehand surgery (FH) consistently ranked last, highlighting substantial accuracy advantages of computer‐assisted techniques in implant placement (Figure 5; Table 6; Table S3).

**TABLE 6:** Ranking of surgical guidance protocols by mean deviation at angular, platform, and apex levels.

Group	Estimate (SE)	95% CIs	Ranking
Angular deviation			
rCAIS	0.48 (0.34)	−0.20 to 1.17	1
dCAIS	1.53 (0.22)	1.08 to 1.98	2
HG‐sCAIS	2.02 (0.42)	1.18 to 2.86	3
FG‐sCAIS	2.32 (0.24)	1.84 to 2.79	4
FH	4.15 (0.31)	3.54 to 4.76	5
Platform deviation			
dCAIS	0.55 (0.06)	0.423–0.687	1
rCAIS	0.57 (0.09)	0.387–0.762	2
FG‐sCAIS	0.72 (0.06)	0.591–0.857	3
HG‐sCAIS	0.95 (0.09)	0.763–1.139	4
FH	1.26 (0.06)	1.135–1.40	5
Apex deviation			
rCAIS	0.45 (0.20)	0.053–0.849	1
dCAIS	0.56 (0.127)	0.313–0.812	2
HG‐sCAIS	0.65 (0.25)	0.155–1.145	3
FG‐sCAIS	1.06 (0.22)	0.650–1.488	4
FH	1.87 (0.187)	1.511–2.247	5

*Note:* Estimates are derived from mixed‐effects network meta‐analysis models. Rankings (1–5) are color‐coded from best (green) to worst (red) performance.

### 3.5 Heterogeneity and Random Effects

Across the three linear mixed‐effects network meta‐regression models, between‐study heterogeneity was generally low to negligible, suggesting that the majority of variability in implant placement accuracy was explained by within‐study differences or accounted for by included covariates. For angular deviation, the estimated between‐study variance (*τ* ^2^) was 0.149 (SD = 0.386), with residual variance (*σ* ^2^) of 5.30 (SD = 2.30), indicating modest heterogeneity. For platform deviation, between‐study variance was minimal (*τ* ^2^ = 0.0065; SD = 0.080), while residual variance was 0.801 (SD = 0.895), reflecting low heterogeneity. For apex deviation, the model estimated no between‐study heterogeneity (*τ* ^2^ = 0), with all variance arising from residual error (*σ* ^2^ = 15.66; SD = 3.96). These values support that the multivariable model specification effectively explained study‐level differences, and that the impact of between‐study variation on outcome estimates was limited.

### 3.6 Transitivity and Consistency

A formal assessment of transitivity showed no systematic differences in key effect modifiers across treatment groups. Consistency testing demonstrated that the RCT‐only networks were largely coherent, although inconsistency was detected for angular deviation; no evidence of disagreement between direct and indirect evidence was observed for platform or apex deviation. As expected, mixed‐design networks (RCTs + observational studies) showed detectable between‐design inconsistency, and therefore their results were interpreted as supportive sensitivity analyses (Supporting Information).

### 3.7 Sensitivity Analysis

Two sensitivity NMAs were performed: (1) RCT‐only, and (2) RCTs plus low‐to‐moderate–risk non‐randomized studies. Across all three modelling strategies (primary arm‐based NMA including all designs, low/moderate–risk‐of‐bias NMA, and RCT‐only frequentist NMA), all guided protocols consistently showed lower angular, platform, and apex deviation than freehand placement. In contrast, differences between guided protocols were less stable: in the primary mixed‐design model, rCAIS and dCAIS tended to rank as most accurate (followed by HG‐sCAIS and FG‐sCAIS), whereas in the RCT‐only networks these pairwise differences were no longer statistically significant, reflecting reduced precision rather than reversal of effect. However, it should be noted that no rCAIS arm was included in the RCT‐only NMA due to lack of eligible study (Supporting Information).

### 3.8 Certainty of Evidence Through CINeMA Approach

Across the 10 evaluated comparisons, the most frequent reasons for downgrading were within‐study bias and reporting bias, both of which reflected the predominance of non‐randomized study designs and incomplete outcome reporting in several included studies. No concerns were identified for indirectness, imprecision, heterogeneity, or incoherence, and no comparison showed evidence of statistical inconsistency in the main model. As a result, despite consistent effect estimates across the network, the overall certainty of evidence remained low due to the methodological limitations inherent to the contributing studies (Table S4).

## 4 Discussion

This systematic review and network meta‐analysis offers a comprehensive evaluation of the accuracy of guided implant surgery techniques (static, dynamic, and robotic) in the context of IIP, compared to conventional freehand placement. The findings affirm previous literature, showing that all guided techniques achieve significantly greater accuracy than FH implant placement. Across all outcome measures, including angular and linear deviation at both the implant platform and apex, guided approaches consistently demonstrated superior performance. Among the techniques analyzed, rCAIS emerged as the most accurate in terms of angular and apical linear deviation. Robustness was confirmed through two sensitivity analyses (RCT‐only; RCT + low‐risk retrospective cohorts), both of which demonstrated comparable effect patterns and rankings as the primary mixed‐design NMA. However, the RCT‐only network excluded the robotic arm entirely, which was a prespecified comparator in our PICOS framework and therefore could not serve as the main analytic model. Nonetheless, consistency diagnostics and effect estimates from all sensitivity analyses are presented in full in the Supporting Information. The sensitivity NMAs restricted to RCTs and to low/moderate–risk studies broadly corroborated the main model: all guided techniques achieved clinically meaningful reductions in deviation compared with freehand across all three outcomes. However, the comparative ranking among guided protocols was less certain in the RCT‐only analyses, where dCAIS, FG‐sCAIS and HG‐sCAIS generally performed similarly within wide confidence intervals. Thus, while our primary arm‐based NMA suggests that rCAIS and dCAIS may offer the lowest deviations, these findings should be interpreted cautiously and as hypothesis‐generating rather than definitive.

A recently published network meta‐analysis on the same topic highlighted the significant improvement in implant position accuracy achieved with guided surgery compared with freehand placement; however, by restricting inclusion to RCTs, that review enhanced the reliability of its conclusions but did not include any study on rCAIS, thereby preventing any assessment of the accuracy of robotic‐assisted implant placement (Schiavon et al. 2025). In line with this, our conclusions regarding robotic‐assisted implant surgery should be interpreted cautiously, since current evidence on rCAIS mainly derives from retrospective studies, resulting in a lower overall certainty of evidence than that supporting static and dynamic navigation systems.

In terms of findings, our analysis revealed statistically significant differences in accuracy among the various guided techniques, a result that was not observed in the NMA by Schiavon et al. (2025). This discrepancy may be explained by the use of less restrictive inclusion criteria regarding study design, which allowed us to include a larger number of patients and implants, thereby increasing the overall statistical power and sensitivity.

Notably, in our analysis no significant differences were observed between rCAIS and dCAIS in terms of platform and apex deviations. These results align with a recent study (Chen et al. 2025), which also suggests that robotic systems offer only marginal improvements in accuracy over dynamic navigation. The limited discrepancy between these two approaches may be attributed to the fact that most included studies were conducted by experienced clinicians with advanced proficiency in guided surgical protocols. In such settings, operators can maintain high levels of manual control and precision, especially during the drilling process, even when using dynamic systems that rely solely on visual guidance without physical control of the handpiece. However, in routine clinical practice, particularly when guided surgery is performed by clinicians in an early stage of their learning curve, robotic systems may offer more substantial advantages. Robotic‐assisted surgery provides complete stabilization of the handpiece during osteotomy preparation, potentially minimizing the risk of deviation due to operator inexperience or hand movement (Bahrami et al. 2024). This is especially critical in IIP, where the empty post‐extraction socket creates a natural void that can cause the drill to deviate or slip, increasing the risk of positional inaccuracy.

The modest differences in accuracy detected between dCAIS and FG‐sCAIS are consistent with recent systematic reviews (Khaohoen et al. 2025; Mahardawi et al. 2025), which compared static and dynamic guided systems for implant placement in healed sites. Both reviews concluded that no significant differences were observed between static and dynamic systems, suggesting that the two techniques exhibit comparable efficacy in enhancing implant placement accuracy under standard conditions.

Although our review identified statistically significant differences in some specific comparisons, these discrepancies may be attributed to the increased technical complexity inherent in post‐extraction sites. As previously discussed, the anatomical characteristics of the socket can cause the drill to deviate toward the path of least resistance. With FG‐sCAIS, such deviations often occur within the tolerances allowed by the surgical sleeve‐handle‐bur system and may not be readily apparent to the clinician due to limited visual access during fully guided static surgery (Raabe et al. 2023). In these scenarios, static guides offer limited real‐time feedback, making it difficult for the operator to detect and correct drilling inaccuracies as they occur. In contrast, dynamic navigation systems continuously monitor the position and trajectory of the drill, providing live feedback and maintaining the operator's awareness of the ideal implant position throughout the procedure (Pellegrino et al. 2021).

Another factor that may have influenced the comparison between static guides and dynamic navigation is the inclusion, within the FG‐CAIS group, of one study (Albiero et al. 2019) that involved IIP in fully edentulous arches using mucosa‐supported surgical templates. These templates have been reported to be less accurate than tooth‐supported guides in several studies (Raico Gallardo et al. 2017; Shi et al. 2023). Nevertheless, given that only a single study of this type was included and that its reported deviations were within the overall range observed in other studies involving tooth‐supported guides, its influence on the overall findings is likely minimal.

An interesting finding from our NMA model was the higher ranking of HG‐sCAIS compared to FG‐sCAIS. However, it is important to note that when translated to clinical practice, a deviation of less than 0.5 degrees/mm in the evaluated parameters is generally considered clinically negligible. When comparing these results to the previous studies comparing fully‐ versus half‐guided static implant placements, Gargallo‐Albiol et al. (2020) and Einsiedel et al. (2024) reported similar findings. While in both studies they discovered significantly better accuracy in FG‐CAIS group, similar to our findings the differences in deviations recorded were clinically negligible (0.17 mm (Einsiedel et al. 2024) and 0.51 mm (Gargallo‐Albiol et al. 2020) inter‐group differences). While further exploration is warranted, the variations in definition of full versus half guided surgery in the included articles should be acknowledged and may have contributed to the observed outcomes.

Although the statistical models employed in this meta‐analysis accounted for the most significant confounding variables, certain limitations must be acknowledged. One major limitation is the inclusion of retrospective cohort and case series study designs, which may lack the rigorous standardization of surgical protocols typically ensured in prospective designs. Additionally, variability in the digital planning software and implant systems used across studies may have introduced heterogeneity that could not be fully controlled, potentially affecting the comparability of outcomes across different interventions. Moreover, the inclusion of single‐arm studies in the model inherently weakens the robustness and reliability of the effect estimates, a limitation that is reflected by the reduced certainty of evidence indicated by the CINeMA assessment.

The outcomes analyzed (angular and linear deviations at the implant platform and apex) were the only parameters consistently reported across the included studies. The heterogeneity in reporting results, combined with the limited number of studies providing separate measurements for mesio‐distal, bucco‐lingual, and corono‐apical components of linear deviation, limited the possibility of performing an evaluation of the precision of each surgical method.

Furthermore, it was not possible to conduct a cost‐effectiveness analysis of the different guided surgery systems, which represents another important aspect for clinical decision‐making that remains to be addressed in future research.

## 5 Conclusions

This systematic review and network meta‐analysis showed that all evaluated guided surgical protocols significantly improve the accuracy of immediate implant placement relative to conventional freehand methods. Across modeling strategies, guided techniques consistently produced lower angular, platform, and apical deviations. Robotic‐assisted surgery and dynamic navigation generally ranked among the most precise modalities in the primary analysis; however, sensitivity analyses limited to RCTs or low–risk‐of‐bias studies revealed reduced precision in pairwise distinctions among guided protocols. Taken together, the evidence supports the clear clinical advantage of guided surgery over freehand placement, while suggesting that the relative superiority of specific guided systems should be interpreted with appropriate caution.

## Author Contributions

**Paolo Nava:** conceptualization, methodology, writing – original draft, data curation, investigation, visualization. **Hamoun Sabri:** conceptualization, methodology, formal analysis, visualization, writing – original draft. **Parham Hazrati:** methodology, investigation, formal analysis, writing – review and editing. **Carlo Nava:** investigation, data curation, writing – review and editing. **Muhammad H. A. Saleh:** writing – review and editing, supervision, project administration. **Hom‐Lay Wang:** writing – review and editing, supervision, project administration. [Correction added on 9 March 2026, after first online publication: The Author Contributions section has been revised.]

## Funding

The authors have nothing to report.

## Conflicts of Interest

The authors declare no conflicts of interest.

## Supporting information


**Data S1:** clr70100‐sup‐0001‐Supinfo.docx.

**Data S2:** clr70100‐sup‐0002‐Supinfo1.docx.

## Data Availability

The data that support the findings of this study are available from the corresponding author upon reasonable request.
