# Supplementary material for: Accuracy of Static, Dynamic, and Robotic Guided Surgery in Immediate Implant Placement: A Systematic Review and Network Meta‐Analysis
Source: Clin Oral Implants Res. 2026 Feb 19;37(5):525–42. doi: 10.1111/clr.70100 (PMC13155306; doi:10.1111/clr.70100)
Supplement: Supplementary file 1 — Data S1: clr70100‐sup‐0001‐Supinfo.docx. [file CLR-37-525-s002.docx]

**SUPPLEMENTARY MATERIAL**

**Accuracy of Static, Dynamic, and Robotic Guided Surgery in Immediate Implant Placement: A Systematic Review and Network Meta-Analysis**

|  |  | **Page** |
| --- | --- | --- |
| **Table S1** | Reasons for article exclusion | **2** |
| **Table S2** | Pairwise comparisons of implant placement accuracy | **3** |
| **Table S3** | Pooled unweighted mean angular, base, and apex deviations | **4** |
|  | **Transitivity assessment** | **4** |
| **Table S4** | Distribution of continuous potential effect modifiers across treatment groups | **5** |
| **Table S5** | Distribution of categorical effect modifiers | **5** |
| **Table** **S6** | Statistical Tests of Transitivity | **6** |
|  | **Sensitivity Analysis** | **7** |
| **Table S7** | Pairwise Comparisons | **8** |
| **Table S8** | Treatment Ranking | **9** |
| **Table S9** | League Table | **9** |
| **Table S10** | Pairwise Comparisons | **10** |
| **Table S11** | Treatment Ranking (P-scores) | **10** |
| **Table S12** | League Table | **10** |
| **Table S13** | Pairwise Comparisons | **11** |
| **Table S14** | Treatment Ranking | **11** |
| **Table S15** | League Table | **11** |
| **Table S16** | RCT-only network meta-analysis for angular deviation | **13** |
| **Table S17** | Ranking table of implant placement protocols based on angular deviation (RCT-only NMA) | **13** |
| **Table S18** | League table of pairwise mean differences in angular deviation (RCT-only NMA) | **13** |
| **Table S19** | RCT-only network meta-analysis for platform deviation (mm) | **13** |
| **Table S20** | League table of pairwise mean differences (mm) in platform deviation among protocols (RCT-only NMA) | **14** |
| **Table S21** | Ranking of implant placement protocols for platform deviation (P-scores; RCT-only NMA) | **14** |
| **Table S22** | Apex deviation – treatment effects vs freehand (FH). (RCT-only NMA) | **14** |
| **Table S23** | Apex deviation – random-effects league table. (RCT-only NMA) | **15** |
| **Table S24** | Apex deviation – ranking by P-scores (RCT-only NMA) | **15** |
| **Table S25** | Confidence in the Evidence (CINeMA Framework) | **16** |
| **Figure S1** | Network geometry diagrams for the sensitivity analyses | **17** |
|  | **Consistency Assessment** | **18** |

**TABLE S1** Reasons for excluding retrieved studies.

| Study | Reason |
| --- | --- |
| Ayman et al. 2021^1^ | Group size not reported (n. of implants) |
| Chen et al., 2025^2^ | Not immediate implantation. |
| Li et al., 2025^3^ | In Chinese |
| Pozzi et al., 2024^4^ | Immediate restoration and not immediate implantation. |
| Shu et al., 2024^5^ | Immediate restoration and not immediate implantation. |
| Pirooz et al., 2023^6^ | Immediate restoration and not immediate implantation. |
| Song et al., 2023^7^ | Cadaver study |
| Zhang et al., 2021^8^ | Immediate restoration and not immediate implantation. |
| Chen et al., 2018^9^ | Cadaver study |

**References**

1. Ayman DM EA, Elkerdawy MW. Evaluation of Accuracy in Computer Guided Versus Free Hand Immediate Implant Placement in Fresh Extraction Sockets: a Randomized Controlled Clinical Trial. *Indian Journal of Public Health Research & Development* 2021;13:131-142.
2. Chen J, Wang Y, Bai Y, et al. Accuracy, Safety, and Efficiency in Robotic-Assisted vs. Freehand Dental Implant Surgery: A 6-Month Follow-Up Randomized Controlled Trial. *Clinical Oral Implants Research* 2025;n/a.
3. Li H, Ma F, Weng J, Du Y, Wu B, Sun F. [Accuracy of dynamic navigation system for immediate dental implant placement]. *Beijing Da Xue Xue Bao Yi Xue Ban* 2025;57:85-90.
4. Pozzi A, Carosi P, Laureti A, et al. Accuracy of navigation guided implant surgery for immediate loading complete arch restorations: Prospective clinical trial. *Clin Implant Dent Relat Res* 2024.
5. Shu Q, Chen D, Wang X, Liu Q, Ge Y, Su Y. Accuracy of flapless surgery using an autonomous robotic system in full-arch immediate implant restoration: A case series. *J Dent* 2024;145:105017.
6. Pirooz P, Atri F, Gholami P, Bayat M. Digital implant placement accuracy: a clinical study on a fully-guided flapless single-unit immediate-loading protocol. *Maxillofacial Plastic and Reconstructive Surgery* 2023;45.
7. Song YW, Park J-Y, Jung J-Y, Kim J-N, Hu K-S, Lee J-S. Does the fixture thread depth affect the accuracy of implant placement during fully guided immediate implant placement?: A human cadaver study. *Clinical Oral Implants Research* 2023;34:116-126.
8. Zhang X, Wang M, Mo A. An alternative method for immediate implant-supported restoration of anterior teeth assisted by fully guided templates: A clinical study. *J Prosthet Dent* 2021;126:636-645.
9. Chen Z, Li J, Sinjab K, Mendonca G, Yu H, Wang H-L. Accuracy of flapless immediate implant placement in anterior maxilla using computer-assisted versus freehand surgery: A cadaver study. *Clinical Oral Implants Research* 2018;29:1186-1194.

**Table S2.** Pairwise comparisons of implant placement accuracy among different guided and freehand techniques

| **Angular Deviation** | | | | |
| --- | --- | --- | --- | --- |
| **Contrast** | **Estimate** | **95% CI** | | **Adjusted p-value (FDR)** |
| dCAIS - rCAIS | 1.05 | 0.06; 2.15 | | **0.0096** |
| dCAIS - FH | -2.62 | -3.33; -1.91 | | **<0.0001** |
| dCAIS - FGsCAIS | -0.79 | -1.27; -0.31 | | **<0.0001** |
| dCAIS - HGsCAIS | -0.49 | -1.87; 0.89 | | 0.3533 |
| rCAIS - FH | -3.67 | -4.89; -2.45 | | **<0.0001** |
| rCAIS - FGsCAIS | -1.84 | -2.94; -0.73 | | **<0.0001** |
| rCAIS - HGsCAIS | -1.54 | -2.94; -0.15 | | **0.0028** |
| FH - FGsCAIS | 1.83 | 1.09; 2.58 | | **<0.0001** |
| FH - HGsCAIS | 2.13 | 0.64; 3.62 | | **0.0001** |
| FGsCAIS - HGsCAIS | 0.30 | -1.09; 1.68 | | 0.5483 |
| **Platform Deviation** | | | | |
| **Contrast** | **Estimate** | **95% CI** | | **Adjusted p-value (FDR)** |
| dCAIS - rCAIS | -0.017 | -0.272; 0.238 | | 0.853 |
| dCAIS - FH | -0.711 | -0.769; -0.652 | | **<0.0001** |
| dCAIS - FGsCAIS | -0.165 | -0.234; -0.096 | | **<0.0001** |
| dCAIS - HGsCAIS | -0.428 | -0.700; -0.156 | | **<0.0001** |
| rCAIS - FH | -0.694 | -0.947; -0.440 | | **<0.0001** |
| rCAIS - FGsCAIS | -0.148 | -0.398; 0.102 | | 0.106 |
| rCAIS - HGsCAIS | -0.411 | -0.672; -0.151 | | **<0.0001** |
| FH - FGsCAIS | 0.546 | 0.468; 0.623 | | **<0.0001** |
| FH - HGsCAIS | 0.282 | 0.011; 0.554 | | **0.0051** |
| FGsCAIS - HGsCAIS | -0.263 | -0.531; -0.005 | | **0.0072** |
| **Apex Deviation** | | | | |
| **Contrast** | **Estimate** | | **95% CI** | **Adjusted p-value (FDR)** |
| dCAIS - rCAIS | -0.120 | | -0.940; 0.699 | 0.6791 |
| dCAIS - FH | -1.370 | | -1.845; -0.895 | **<.0001** |
| dCAIS - FGsCAIS | -0.547 | | -1.157; -0.063 | **0.0293** |
| dCAIS - HGsCAIS | -0.819 | | -1.801; -0.163 | **0.0374** |
| rCAIS - FH | -1.250 | | -2.102; -0.397 | **0.0002** |
| rCAIS - FGsCAIS | -0.427 | | -1.242; 0.389 | 0.1749 |
| rCAIS - HGsCAIS | -0.698 | | -1.612; 0.216 | 0.0515 |
| FH - FGsCAIS | 0.823 | | 0.113; 1.533 | **0.0039** |
| FH - HGsCAIS | 0.551 | | -0.466; 1.569 | 0.1749 |
| FGsCAIS - HGsCAIS | -0.272 | | -1.266; 0.722 | 0.4816 |

**Table S3.** Pooled unweighted mean angular, base, and apex deviations (in mm or degrees) for each surgical guidance group. Mean values, standard deviations (SD), and sample sizes (N) are reported separately for each group and each outcome. These values represent simple pooled statistics without model adjustment and were used for descriptive reference in the appendix.

| **Outcome** | **GROUP** | **Mean** | **SD** | **N** |
| --- | --- | --- | --- | --- |
| **Angular Deviation** | dCAIS | 1.94 | 0.945 | 6 |
|  | rCAIS | 1.39 | 0.364 | 5 |
|  | FH | 5.08 | 1.51 | 5 |
|  | FG-sCAIS | 2.72 | 1.42 | 8 |
|  | HG-sCAIS | 2.54 | 1.09 | 4 |
| **Platform Deviation** | dCAIS | 0.785 | 0.216 | 6 |
|  | rCAIS | 0.674 | 0.142 | 5 |
|  | FH | 1.34 | 0.117 | 4 |
|  | FG-sCAIS | 0.93 | 0.255 | 7 |
|  | HG-sCAIS | 0.927 | 0.466 | 4 |
| **Apex Deviation** | dCAIS | 0.897 | 0.324 | 6 |
|  | rCAIS | 0.694 | 0.182 | 5 |
|  | FH | 1.82 | 0.372 | 4 |
|  | FG-sCAIS | 1.34 | 0.658 | 7 |
|  | HG-sCAIS | 1.22 | 0.727 | 4 |

In addition to the mixed-effects modeling presented in the main results, we computed unweighted pooled mean values, standard deviations, and sample sizes for each outcome measure (angular deviation, platform deviation, and apex deviation) within each surgical guidance group. These values serve to provide descriptive insight into the central tendency and spread observed outcomes without statistical adjustment. While not adjusted for study-level clustering or covariates such as anatomical location or study design, these pooled descriptive statistics offer useful baseline comparisons and support interpretability of the adjusted marginal means reported in the main analysis.

**Transitivity Assessment**

To evaluate the plausibility of the transitivity assumption, we compared the distribution of key potential effect modifiers across all treatment groups (dCAIS, rCAIS, FG-sCAIS, FH, HG-sCAIS). Continuous effect modifiers (Maxilla percentage, Anterior percentage, number of patients per arm, and number of implants per arm) showed no statistically significant differences between groups (all p > 0.13, Kruskal–Wallis). Categorical characteristics likewise showed no evidence of imbalance, including study design (p = 0.45), risk of bias (p = 0.59), and presence of open-ended sites (p = 0.67).

Overall, the distributions of anatomical, methodological, and study-level factors were broadly comparable across treatments, supporting the assumption that the included studies are jointly exchangeable and therefore fulfilling the transitivity requirement for network meta-analysis.

**Table S4.** Distribution of continuous potential effect modifiers across treatment groups

| **Group** | **Maxilla (%)** | **Anterior (%)** | **N Patients** | **N Implants** |
| --- | --- | --- | --- | --- |
| **dCAIS** | n=6; mean 83.3; SD 40.8; median 100; IQR 40.8–100 | n=6; mean 66.7; SD 36.3; median 83.3; IQR 50–100 | n=6; mean 12; SD 1.4; median 12; range 10–14 | n=6; mean 22; SD 2.1; median 22; range 20–24 |
| **FG-sCAIS** | n=8; mean 72.5; SD 34.5; median 80; IQR 47.5–100 | n=8; mean 66.5; SD 32.7; median 50; IQR 50–100 | n=8; mean 10; SD 2.6; median 10; range 7–15 | n=8; mean 60; SD 30; median 50; range 30–100 |
| **FH** | n=5; mean 65.5; SD 42.9; median 82.5; IQR 45–100 | n=5; mean 50; SD 50; median 50; IQR 0–100 | n=5; mean 32; SD 26; median 32 | n=5; mean 40; SD 30; median 40 |
| **HG-sCAIS** | n=4; mean 71.7; SD 29.0; median 86.9 | n=4; mean 75; SD 5; median 75 | n=4; mean 19; SD 11 | n=4; mean 23; SD 12 |
| **rCAIS** | n=5; mean 68.7; SD 31.4; median 66.7 | n=5; mean 70; SD 4; median 70 | n=5; mean 21; SD 15 | n=5; mean 33; SD 10 |

**Table S5.** Distribution of categorical effect modifiers across treatment groups.

| **Study design** | | | |
| --- | --- | --- | --- |
| **Group** | **Case Series** | **RCT** | **Retrospective** |
| dCAIS | 1 | 3 | 2 |
| FG-sCAIS | 1 | 3 | 4 |
| FH | 0 | 3 | 2 |
| HG-sCAIS | 1 | 1 | 2 |
| rCAIS | 0 | 0 | 5 |
| **Risk of bias** | | | |
| **Group** | **High** | **Low** | **Moderate** |
| dCAIS | 0 | 4 | 2 |
| FG-sCAIS | 1 | 3 | 4 |
| FH | 0 | 3 | 2 |
| HG-sCAIS | 0 | 2 | 2 |
| **Open ended sites** | | | |
| **Group** | **No** | **Yes** |  |
| dCAIS | 4 | 2 |  |
| FG-sCAIS | 6 | 2 |  |
| FH | 3 | 2 |  |
| HG-sCAIS | 4 | 0 |  |
| rCAIS | 3 | 2 |  |

**Table** **S6**. Statistical Tests of Transitivity.

| **Continuous variables (Kruskal–Wallis)** | | | |  |
| --- | --- | --- | --- | --- |
| **Modifier** | **KW χ²(df=4)** | **p-value** | **Interpretation** |  |
| Maxilla % | 2.85 | **0.584** | No difference across groups |  |
| Anterior % | 0.96 | **0.915** | No difference across groups |  |
| N Patients | 7.05 | **0.134** | No difference across groups |  |
| N Implants | 5.56 | **0.235** | No difference across groups |  |
|  |  |  |  |  |
| **Categorical variables (χ² tests)** | | | |  |
| **Modifier** | **χ²** | **df** | **p-value** | **Interpretation** |
| Study Design | 7.82 | 8 | **0.452** | No imbalance |
| Risk of Bias | 6.54 | 8 | **0.587** | No imbalance |
| Open-Ended Sites | 2.36 | 4 | **0.671** | No imbalance |

**Sensitivity Analysis**

- **Network meta-analysis including RCTs and low-moderate non-randomized studies**

1. **Angular Deviation**

After restricting the analysis to low- and moderate–risk-of-bias non-randomized controlled clinical trials plus RCTs, the ranking pattern remained consistent with the primary model (**Tables S7-S9)**. rCAIS demonstrated the highest accuracy (P-score = 0.863), followed by dCAIS, HG-sCAIS, FG-sCAIS, and with FH performing worst (P-score = 0.001). Several key pairwise comparisons remained statistically significant. Both dCAIS and FG-sCAIS continued to outperform FH (dCAIS vs FH: MD = −3.43°; 95% CI −5.66 to −1.20; p = 0.0026; FG vs FH: MD = −3.46°; 95% CI −5.37 to −1.55; p = 0.0004). Importantly, FH performed significantly worse than both HG-sCAIS and rCAIS (FH vs HG-sCAIS: MD = 3.52°; 95% CI 0.20 to 6.84; p = 0.04; FH vs rCAIS: MD = 5.00°; 95% CI 1.94 to 8.06; p = 0.001).

No statistically significant differences were observed among the guided approaches themselves (e.g., dCAIS vs FG-sCAIS, dCAIS vs rCAIS, FG-sCAIS vs HG-sCAIS, FG-sCAIS vs rCAIS, HG-sCAIS vs rCAIS; all p > 0.30), indicating comparable performance among the guided modalities within this evidence set.

For angular deviation, heterogeneity and/or inconsistency was considerable (τ² = 2.67, τ = 1.63°, I² = 91.7%), with a highly significant total Q (Q = 60.01, df = 5, p < 0.0001). This was driven mainly by between-design inconsistency (Q_between = 57.27, df = 4, p < 0.0001), while within-design heterogeneity was not statistically significant (Q_within = 2.74, df = 1, p = 0.098).

1. **Platform Deviation**

When the analysis was restricted to low- and moderate–risk-of-bias studies and RCTs, the treatment ranking remained consistent with the main model. dCAIS showed the highest precision at the platform level, achieving the top P-score (0.950), followed by rCAIS, FG-sCAIS, and HG-sCAIS, whereas free-hand placement performed the worst (P-score = 0.001) (**Tables S10-S12**).

Multiple pairwise comparisons remained statistically significant. dCAIS outperformed FG-sCAIS, FH, and HG-sCAIS, demonstrating smaller platform deviation against all three (dCAIS vs FG-sCAIS: MD = −0.176 mm; p < 0.001; dCAIS vs FH: MD = −0.728 mm; p < 0.001; dCAIS vs HG-sCAIS: MD = −0.479 mm; p < 0.001). FG-sCAIS also performed significantly better than FH and HG-sCAIS (FG-sCAIS vs FH: MD = −0.552 mm; p < 0.001; FG-sCAIS vs HG-sCAIS: MD = −0.303 mm; p = 0.005).

Across the other guided comparisons, rCAIS outperformed FH and HG-sCAIS (FH vs rCAIS: MD = 0.652 mm; p < 0.001; HG-sCAIS vs rCAIS: MD = 0.404 mm; p < 0.001), while no significant differences were detected among the guided modalities themselves (e.g., dCAIS vs rCAIS, FG-sCAIS vs rCAIS; all p > 0.25). The network demonstrated no detectable inconsistency (I² = 0%).

For platform deviation, between-study heterogeneity and network inconsistency were negligible (τ² = 0, τ = 0, I² = 0%), and neither the total Q (Q = 3.97, df = 4, p = 0.41), the within-design component (Q_within = 0.94, df = 1, p = 0.33), nor the between-design component (Q_between = 3.03, df = 3, p = 0.39) indicated any meaningful inconsistency.

Overall, the sensitivity analysis reinforces the superiority of all guided approaches over free-hand implantation and confirms dCAIS as the most accurate system for minimizing platform deviation.

1. **Apex Deviation**

When restricting the analysis to low- and moderate–risk-of-bias studies, the ranking pattern remained aligned with the primary model. dCAIS demonstrated the highest apex-level accuracy (P-score = 0.952), followed by rCAIS, FG-sCAIS, and HG-sCAIS, while free-hand placement (FH) performed worst (**Tables S13-S15**).

Multiple pairwise comparisons remained statistically significant. dCAIS showed significantly lower apex deviation than FG-sCAIS, FH, and HG-sCAIS (dCAIS vs FG: MD = −0.568 mm; p = 0.005; dCAIS vs FH: MD = −1.427 mm; p < 0.001; dCAIS vs HG: MD = −1.144 mm; p < 0.001). FG-sCAIS also outperformed FH (MD = −0.859 mm; p < 0.001), though FG-sCAIS vs HG-sCAIS and FG-sCAIS vs rCAIS did not reach statistical significance.

Free-hand placement was consistently inferior: FH performed significantly worse than HG-sCAIS and rCAIS (FH vs HG-sCAIS: MD = 0.569 mm; p = 0.015; FH vs rCAIS: MD = 1.144 mm; p < 0.001). HG-sCAIS also showed significantly greater deviation than rCAIS (HG-sCAIS vs rCAIS: MD = 0.576 mm; p = 0.03).

For apex deviation, overall variability was moderate to substantial (τ² = 0.07, τ = 0.27 mm, I² = 69.4%), with a significant total Q (Q = 13.07, df = 4, p = 0.011). This pattern was mainly explained by between-design inconsistency (Q_between = 10.24, df = 3, p = 0.017), whereas within-design heterogeneity did not reach statistical significance (Q_within = 2.83, df = 1, p = 0.093).

Overall, the sensitivity analysis confirms the superiority of all guided methods over free-hand placement at the apical level, with dCAIS maintaining the most consistent performance across studies. Unlike the base-deviation network, apex deviation revealed significant inconsistency across designs (Q_between = 10.24, p = 0.016).

**Table S7.** Pairwise Comparisons (Random-Effects Model) for the outcome of angular deviation. (Negative MD = better accuracy than comparator).

| **Comparison** | **MD (°)** | **95% CI** | **p-value** |
| --- | --- | --- | --- |
| dCAIS vs FG-sCAIS | 0.03 | −2.08 to 2.14 | 0.95 |
| dCAIS vs FH | **−3.43** | **−5.66 to −1.20** | **0.0026** |
| dCAIS vs HG-sCAIS | 0.10 | −2.74 to 2.93 | 0.34 |
| dCAIS vs rCAIS | 1.57 | −2.04 to 5.18 | 0.39 |
| FG-sCAIS vs FH | **−3.46** | **−5.37 to −1.55** | **0.0004** |
| FG-sCAIS vs HG-sCAIS | 0.07 | −2.57 to 2.71 | 0.96 |
| FG-sCAIS vs rCAIS | 1.54 | −1.74 to 4.82 | 0.35 |
| FH vs HG-sCAIS | **3.52** | **0.20 to 6.84** | **0.04** |
| FH vs rCAIS | **5.00** | **1.94 to 8.06** | **0.001** |
| HG-sCAIS vs rCAIS | 1.48 | −1.57 to 4.53 | 0.34 |

**Table S8.** Treatment Ranking (P-scores). Higher P-score = greater probability of being most accurate (lowest angular deviation).

| **Treatment** | **P-score (random)** |
| --- | --- |
| **rCAIS** | **0.863** |
| **dCAIS** | **0.555** |
| HG-sCAIS | 0.542 |
| FG-sCAIS | 0.539 |
| **FH** | **0.001** |

**Table S9.** League Table (Random-Effects Model). (Rows = treatment A vs columns = treatment B; positive = A worse than B)

|  | **dCAIS** | **FG-sCAIS** | **FH** | **HG-sCAIS** | **rCAIS** |
| --- | --- | --- | --- | --- | --- |
| **dCAIS** | — | 0.03  (−2.08, 2.14) | **−3.43**  **(−5.66, −1.20)** | 0.10  (−2.74, 2.93) | 1.57  (−2.04, 5.18) |
| **FG-sCAIS** | — | — | **−3.46**  **(−5.37, −1.55)** | 0.06  (−2.13, 2.26) | 1.54  (−1.74, 4.82) |
| **FH** | — | — | — | **3.52**  **(0.20, 6.84)** | **5.00**  **(1.94, 8.06)** |
| **HG-sCAIS** | — | — | — | — | 1.48  (−1.78, 4.74) |
| **rCAIS** | — | — | — | — | — |

**Table S10.** Pairwise Comparisons (Random-Effects Model). Pairwise comparisons of platform deviation (mm) among implant placement modalities, derived from the random-effects sensitivity analysis including only low- and moderate-risk-of-bias studies.

| **Comparison** | **MD (mm)** | **95% CI** | **p-value** |
| --- | --- | --- | --- |
| dCAIS vs FG-sCAIS | **−0.176** | −0.214 to −0.138 | **<0.001** |
| dCAIS vs FH | **−0.728** | −0.770 to −0.685 | **<0.001** |
| dCAIS vs HG-sCAIS | **−0.479** | −0.662 to −0.296 | **<0.001** |
| dCAIS vs rCAIS | −0.075 | −0.246 to 0.096 | 0.39 |
| FG-sCAIS vs FH | **−0.552** | −0.597 to −0.507 | **<0.001** |
| FG-sCAIS vs HG-sCAIS | **−0.303** | −0.479 to −0.127 | **0.005** |
| FG-sCAIS vs rCAIS | 0.100 | −0.080 to 0.280 | 0.27 |
| FH vs HG-sCAIS | **0.249** | 0.075 to 0.423 | **0.005** |
| FH vs rCAIS | **0.652** | 0.479 to 0.826 | **<0.001** |
| HG-sCAIS vs rCAIS | **0.404** | 0.243 to 0.564 | **<0.001** |

**Table S11. Treatment Ranking (P-scores).** P-score–based ranking of implant placement modalities for platform deviation (lower deviation = better accuracy) in the low-/moderate-risk-of-bias sensitivity analysis.

| **Treatment** | **P-score (random)** |
| --- | --- |
| **dCAIS** | **0.950** |
| **rCAIS** | **0.767** |
| FG-sCAIS | 0.533 |
| HG-sCAIS | 0.250 |
| **FH** | **0.001** |

**Table S12. League Table (Random-Effects Model).** League table presenting all pairwise random-effects comparisons of platform deviation among implant placement modalities. Each cell shows the mean difference (MD, mm) with 95% confidence intervals; negative values indicate lower deviation (better accuracy) for the row treatment compared to the column treatment.

|  | **dCAIS** | **FG-sCAIS** | **FH** | HG-sCAIS | **rCAIS** |
| --- | --- | --- | --- | --- | --- |
| **dCAIS** | — | −0.18  (−0.21, −0.14) | −0.73  (−0.77, −0.69) | −0.48  (−0.66, −0.30) | −0.08  (−0.25, 0.10) |
| FG-sCAIS | — | — | −0.55  (−0.60, −0.50) | −0.30  (−0.48, −0.13) | 0.10  (−0.08, 0.28) |
| **FH** | — | — | — | 0.25  (0.07, 0.42) | 0.65  (0.48, 0.83) |
| HG-sCAIS | — | — | — | — | 0.39  (0.22, 0.56) |
| **rCAIS** | — | — | — | — | — |

**Table S13.** Pairwise Comparisons (Random-Effects Model). Pairwise random-effects comparisons of apex deviation (mm) among implant placement modalities in the low-/moderate-risk-of-bias sensitivity analysis. Negative values indicate smaller deviation (higher accuracy) for the row treatment.

| **Comparison** | **MD (mm)** | **95% CI** | **p-value** |
| --- | --- | --- | --- |
| dCAIS vs FG-sCAIS | **−0.568** | −0.959 to −0.178 | **0.005** |
| dCAIS vs FH | **−1.427** | −1.827 to −1.027 | **<0.001** |
| dCAIS vs HG-sCAIS | **−1.144** | −1.676 to −0.613 | **<0.001** |
| dCAIS vs rCAIS | −0.286 | −0.912 to 0.341 | 0.37 |
| FG-sCAIS vs FH | **−0.859** | −1.270 to −0.448 | **<0.001** |
| FG-sCAIS vs HG-sCAIS | −0.290 | −0.739 to 0.158 | 0.20 |
| FG-sCAIS vs rCAIS | 0.29 | −0.324 to 0.890 | 0.34 |
| FH vs HG-sCAIS | **0.569** | 0.113 to 1.025 | **0.015** |
| FH vs rCAIS | **1.144** | 0.613 to 1.676 | **<0.001** |
| HG-sCAIS vs rCAIS | **0.576** | 0.046 to 1.106 | **0.03** |

**Table S14.** Treatment Ranking. Ranking of implant placement modalities for apex deviation based on P-scores (random-effects model). Higher P-scores indicate greater accuracy.

| **Treatment** | **P-score (random)** |
| --- | --- |
| **dCAIS** | **0.952** |
| **rCAIS** | **0.749** |
| FG-sCAIS | 0.519 |
| HG-sCAIS | 0.278 |
| **FH** | **0.002** |

**Table S15.** League Table (Random-Effects Model). League table displaying all pairwise random-effects comparisons for apex deviation. Values represent mean differences (MD, mm) with 95% confidence intervals; negative values favor the row treatment.

|  | **dCAIS** | **FG-sCAIS** | **FH** | **HG-sCAIS** | **rCAIS** |
| --- | --- | --- | --- | --- | --- |
| **dCAIS** | — | −0.57  (−0.96, −0.18) | −1.49  (−1.92, −1.05) | −0.86  (−1.38, −0.33) | −0.28  (−0.91, 0.35) |
| **FG-sCAIS** | — | — | −0.89  (−1.43, −0.35) | −0.29  (−0.74, 0.16) | 0.29  (−0.32, 0.89) |
| **FH** | — | — | — | 0.54  (−0.04, 1.12) | 1.14  (0.61, 1.68) |
| **HG-sCAIS** | — | — | — | — | 0.59  (0.03, 1.15) |
| **rCAIS** | — | — | — | — | — |

**RCT-only network meta-analysis**

1. **Angular Deviation**

In the RCT-only contrast-based network meta-analysis (5 randomized trials comparing FH, FG-sCAIS, HG-sCAIS, and dCAIS), all guided protocols showed significantly lower angular deviation than freehand placement. Compared with FH, the random-effects model estimated a reduction of 3.23° for dCAIS (MD –3.23°, 95% CI –5.23 to –1.24; p = 0.0015), 4.35° for FG-sCAIS (MD –4.35°, 95% CI –6.63 to –2.07; p = 0.0002), and 6.11° for HG-sCAIS (MD –6.11°, 95% CI –10.76 to –1.45; p = 0.0101). P-scores based on the random-effects NMA ranked HG-sCAIS as most accurate (P-score 0.89), followed by FG-sCAIS (0.68) and dCAIS (0.43), whereas FH consistently ranked last (0.00). Between-study heterogeneity was substantial (τ² = 1.90; I² = 83.1%), and the design-by-treatment interaction test indicated some inconsistency in this small network (Q_between = 11.32, p = 0.0008), although all guided protocols consistently outperformed freehand placement in terms of angular accuracy. No RCTs involving robotic-assisted guidance were available; therefore, rCAIS could not be included in this RCT-only network and was evaluated only in the mixed-design analyses (**Tables S16-S18)**.

1. **Platform Deviation**

In the RCT-only network for platform deviation, all guided protocols showed reduced deviation compared with freehand placement. Random-effects mean differences (MD) versus FH were –0.70 mm (95% CI: –0.74 to –0.66; p < 0.0001) for dCAIS, –0.77 mm (95% CI: –1.14 to –0.40; p < 0.0001) for FG-sCAIS, and –0.69 mm (95% CI: –1.43 to 0.06; p = 0.0699) for HG-sCAIS. Although the HG-sCAIS comparison did not reach conventional statistical significance, the direction of effect was consistent with the other guided protocols and favored lower platform deviation relative to FH.

Between-study heterogeneity and incoherence were negligible (τ² = 0; I² = 0%; Q_total = 0.77, p = 0.38), indicating a statistically coherent and homogeneous RCT network. P-score–based rankings (random-effects) suggested that FG-sCAIS had the highest probability of being the most accurate for platform deviation (P-score 0.75), followed by dCAIS (0.62) and HG-sCAIS (0.62), whereas FH consistently ranked last (P-score 0.01) **(Tables S19-S21)**.

1. **Apex Deviation**

Restricting the analysis to RCTs resulted in a four-node network (dCAIS, FG-sCAIS, FH, HG-sCAIS) without multi-arm loops. Across studies, apex deviation values were generally lower for guided protocols relative to freehand placement (**Tables S22-24)**.

dCAIS demonstrated significantly lower apex deviation compared to FH (MD −0.853 mm; 95% CI −0.988 to −0.719; p < 0.001). FG-sCAIS similarly outperformed FH (MD −0.533 mm; 95% CI −0.958 to −0.109; p = 0.014). The comparison between dCAIS and FG-sCAIS showed no significant difference (MD −0.320 mm; p = 0.12), indicating comparable performance between these two guided systems. Estimates involving HG-sCAIS were imprecise due to limited direct evidence, and no statistically significant differences were observed in these comparisons. The random-effects league table showed consistent directional trends favoring guided approaches over FH. dCAIS and FG-sCAIS yielded substantially lower apex deviation relative to FH, while comparisons involving HG-sCAIS had wide confidence intervals reflecting sparse data. Across all available pairwise estimates, negative MD values consistently favored the column treatment (i.e., lower deviation).

According to P-scores, HG-sCAIS ranked highest (0.81), followed closely by dCAIS (0.77). FG-sCAIS showed intermediate performance (0.41), and FH ranked lowest (0.02), consistent with both the pairwise effect estimates and overall model results.

The RCT-only apex deviation model showed no detectable between-study heterogeneity (τ² = 0; I² = 0%) and no evidence of inconsistency (Q = 0.59, p = 0.44), indicating that direct and indirect evidence were fully coherent across the network.

**Table S16.** RCT-only network meta-analysis for angular deviation (°). Negative mean differences indicate lower deviation (better accuracy) for the guided protocol compared with freehand (FH). Random-effects frequentist NMA, FH used as reference.

| **Comparison** | **MD (°)** | **95% CI (°)** | **p-value*** |
| --- | --- | --- | --- |
| dCAIS vs FG-sCAIS | 1.12 | −1.19 to 3.42 | 0.34 |
| dCAIS vs FH | −3.23 | −5.23 to −1.24 | **0.002** |
| dCAIS vs HG-sCAIS | 2.88 | −1.79 to 7.54 | 0.23 |
| FG-sCAIS vs FH | −4.35 | −6.63 to −2.07 | **<0.001** |
| FG-sCAIS vs HG-sCAIS | 1.76 | −2.30 to 5.82 | 0.40 |
| FH vs HG-sCAIS | 6.11 | 1.45 to 10.76 | **0.01** |

**Table S17.** Ranking table of implant placement protocols based on angular deviation in the RCT-only NMA (random-effects model). Higher P-scores indicate a higher probability of being the most accurate (lowest angular deviation).

| **Treatment** | **P-score (random)** | **Rank** |
| --- | --- | --- |
| HG-sCAIS | 0.895 | 1 |
| FG-sCAIS | 0.676 | 2 |
| dCAIS | 0.428 | 3 |
| FH | 0.002 | 4 |

**Table S18.** League table of pairwise mean differences in angular deviation (°) between protocols in the RCT-only network (random-effects model). Values represent the mean difference (row – column); negative values favor the column treatment (lower angular deviation).

| **Row \ Column** | **dCAIS** | **FG** | **FH** | **HG** |
| --- | --- | --- | --- | --- |
| **dCAIS** | – | – | – | – |
| **FG-sCAIS** | 1.12 [-1.19; 3.42] | – | – | – |
| **FH** | **–3.23 [-5.23; –1.24]** | **–4.35 [-6.63; –2.07]** | – | – |
| **HG-sCAIS** | 2.88 [-1.79; 7.54] | 1.76 [-2.30; 5.82] | **6.11 [1.45; 10.80]** | – |

**Table S19.** RCT-only network meta-analysis for platform deviation (mm) – guided protocols vs freehand (FH). Negative values indicate lower deviation (better accuracy).

| **Comparison** | **MD (mm)** | **95% CI (mm)** | **p-value** |
| --- | --- | --- | --- |
| **dCAIS vs FG-sCAIS** | 0.07 | −0.30 to 0.44 | 0.71 |
| **dCAIS vs FH** | −0.70 | −0.74 to −0.66 | **<0.001** |
| **dCAIS vs HG-sCAIS** | −0.01 | −0.75 to 0.73 | 0.98 |
| **FG-sCAIS vs FH** | −0.77 | −1.14 to −0.40 | **<0.001** |
| **FG-sCAIS vs HG-sCAIS** | −0.08 | −0.73 to 0.57 | 0.81 |
| **FH vs HG** | 0.69 | −0.06 to 1.43 | 0.07 |

**Table S20.** League table of pairwise mean differences (mm) in platform deviation among protocols (RCT-only, random-effects). Negative values favor the column treatment (lower deviation).

| **Row \ Column** | **dCAIS** | **FG-sCAIS** | **FH** | **HG-sCAIS** |
| --- | --- | --- | --- | --- |
| **dCAIS** | — | — | — | — |
| **FG-sCAIS** | 0.07  (−0.30 to 0.44) | — | — | — |
| **FH** | −0.70  (−0.74 to −0.66)* | −0.77  (−1.14 to −0.40)* | — | — |
| **HG-sCAIS** | −0.01  (−0.75 to 0.73) | −0.08  (−0.73 to 0.57) | 0.69  (−0.06 to 1.43) | — |

**Table S21.** Ranking of implant placement protocols based on P-scores from the RCT-only network meta-analysis for platform deviation (higher P-scores indicate a higher probability of being the most accurate, i.e., lowest deviation).

| **Treatment** | **P-score (random)** | **Rank (random)** |
| --- | --- | --- |
| FG-sCAIS | 0.75 | 1 |
| dCAIS | 0.62 | 2 |
| HG-sCAIS | 0.62 | 3 |
| FH | 0.01 | 4 |

**Table S22.** RCT-only NMA: Apex deviation – treatment effects vs freehand (FH). Negative MD values indicate lower apex deviation (higher accuracy) compared to FH.

| **Comparison** | **MD (mm)** | **95% CI (mm)** | **p-value** |
| --- | --- | --- | --- |
| dCAIS vs FG-sCAIS | −0.320 | −0.723 to 0.082 | 0.12 |
| dCAIS vs FH | −0.853 | −0.988 to −0.719 | **<0.001** |
| dCAIS vs HG-sCAIS | 0.210 | −0.974 to 1.394 | 0.73 |
| FG-sCAIS vs FH | −0.533 | −0.958 to −0.109 | **0.014** |
| FG-sCAIS vs HG-sCAIS | 0.530 | −0.583 to 1.643 | 0.35 |
| FH vs HG-sCAIS | 1.063 | −0.128 to 2.255 | 0.08 |

**Table S23.** RCT-only NMA: Apex deviation – random-effects league table. Cells show MD in apex deviation (mm) with 95% CI. Negative values favor the column treatment (lower deviation).

|  | **dCAIS** | **FG-sCAIS** | **FH** | **HG-sCAIS** |
| --- | --- | --- | --- | --- |
| **dCAIS** | – | - | – | – |
| **FG-sCAIS** | −0.32  (−0.72 to 0.08) | – | – | – |
| **FH** | −0.85  (−0.99 to −0.72)* | −0.53  (−0.96 to −0.11)* | – | – |
| **HG-sCAIS** | 0.21  (−0.97 to 1.39) | 0.53  (−0.58 to 1.64) | 1.06  (−0.13 to 2.25) | – |

**Table S24.** RCT-only NMA: Apex deviation – ranking by P-scores. Higher P-scores indicate better performance (lower apex deviation).

| **Rank** | **Treatment** | **P-score (random)** |
| --- | --- | --- |
| 1 | HG-sCAIS | 0.81 |
| 2 | dCAIS | 0.77 |
| 3 | FG-sCAIS | 0.41 |
| 4 | FH | 0.02 |

**Table S25.** Confidence in the Evidence from Network Meta-Analysis Based on CINeMA Framework. Summary of confidence ratings for each pairwise comparison based on six domains: within-study bias, reporting bias, indirectness, imprecision, heterogeneity, and incoherence. Ratings were derived using the CINeMA (Confidence in Network Meta-Analysis) methodology. Confidence levels (high or moderate) and reasons for downgrading are reported accordingly.

| **Comparison** | **N. of studies** | **Within-study bias** | **Reporting bias** | **Indirectness** | **Imprecision** | **Heterogeneity** | **Incoherence** | **Confidence rating** | **Reason(s) for downgrading** |
| --- | --- | --- | --- | --- | --- | --- | --- | --- | --- |
| **FG-sCAIS:FH** | 2 | Some concerns | Some concerns | No concerns | No concerns | No concerns | No concerns | Low | ["Within-study bias","Reporting bias"] |
| **FG-sCAIS:HG-sCAIS** | 2 | No concerns | Some concerns | No concerns | No concerns | No concerns | No concerns | Low | ["Within-study bias","Reporting bias"] |
| **dCAIS:FG-sCAIS** | 2 | No concerns | Some concerns | No concerns | No concerns | No concerns | No concerns | Low | ["Within-study bias","Reporting bias"] |
| **FH:HG-sCAIS** | 1 | No concerns | Some concerns | No concerns | No concerns | No concerns | No concerns | Low | ["Within-study bias","Reporting bias"] |
| **dCAIS:FH** | 3 | Some concerns | Some concerns | No concerns | No concerns | No concerns | No concerns | Low | ["Within-study bias","Reporting bias"] |
| **FH:rCAIS** | 1 | No concerns | Some concerns | No concerns | No concerns | No concerns | No concerns | Low | ["Within-study bias","Reporting bias"] |
| **HG-sCAIS:rCAIS** | 1 | No concerns | Some concerns | No concerns | No concerns | No concerns | No concerns | Low | ["Within-study bias","Reporting bias"] |
| **FG-sCAIS:rCAIS** | 0 | No concerns | Some concerns | No concerns | No concerns | No concerns | No concerns | Low | ["Within-study bias","Reporting bias"] |
| **dCAIS:HG-sCAIS** | 0 | No concerns | Some concerns | No concerns | No concerns | No concerns | No concerns | Low | ["Within-study bias","Reporting bias"] |
| **dCAIS:rCAIS** | 0 | No concerns | Some concerns | No concerns | No concerns | No concerns | No concerns | Low | ["Within-study bias","Reporting bias"] |

**Figure S1**. Network geometry diagrams for the sensitivity analyses. The upper panel illustrates the RCT-only networks for angular, platform, and apex deviation, plotted using an identical coordinate layout to enable side-by-side comparison. The lower panel presents the expanded networks including RCTs and low–risk-of-bias retrospective studies. Edge thickness denotes the number of available direct comparisons, and shaded polygons indicate closed loops contributing indirect evidence. Nodes represent implant placement techniques: FH, FG-sCAIS (FG), HG-sCAIS (HG), dCAIS, and rCAIS.

**
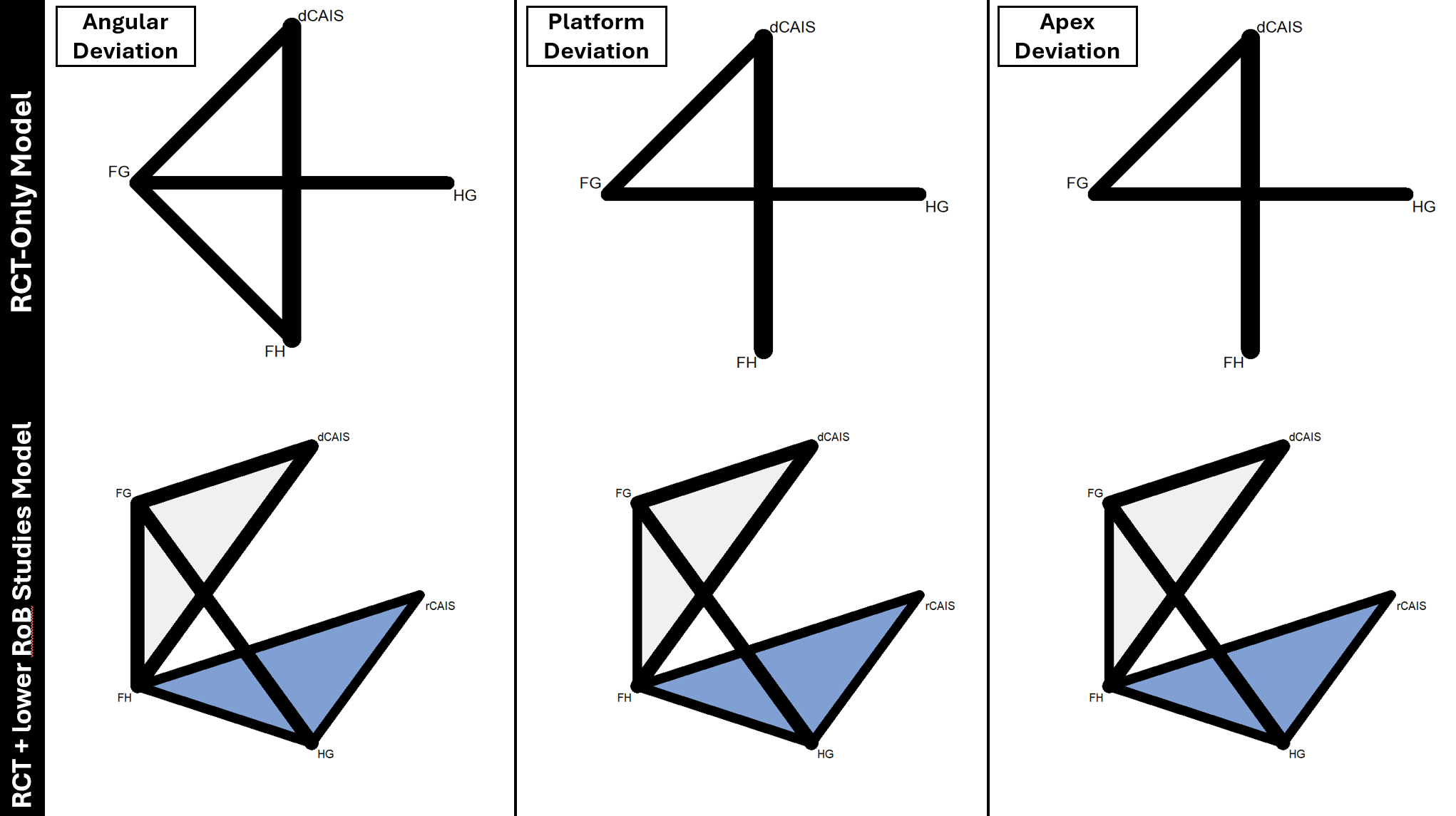
Consistency Assessment (RCT-only NMA)**

In the RCT-only networks for angular, platform, and apical deviation, no multi-arm or closed loops were present; therefore, node-splitting procedures were not estimable. Global design-by-treatment interaction testing showed no evidence of inconsistency within study designs (e.g., angular deviation: Q= 0.50, p = 0.48), indicating excellent agreement between the available direct comparisons. As expected for a sparse, loop-free network, between-design tests are not applicable, and all SIDE comparisons showed identical direct and indirect estimates (where indirect evidence existed), confirming network coherence.

**Consistency Assessment (RCT + Low/Moderate-Risk Non-Randomized Studies)**

When RCTs and low/moderate-risk non-randomized studies were combined, between-design inconsistency was detected for angular deviation (Q= 57.27, p < 0.0001), while platform and apical deviation showed no such inconsistency. This finding reflects structural differences across study designs (e.g., case selection, imaging modality, operator variability), which is expected when mixing heterogeneous study types. Importantly, these design-level differences are explicitly accounted for in the primary arm-based model, which incorporates study design and Study_ID as covariates/random effects. The consistency assessment therefore complements—not contradicts—the main analytical framework by confirming design-related variability that is already modelled in the primary analysis.
